# ATP-responsive biomolecular condensates tune bacterial kinase signaling

**DOI:** 10.1126/sciadv.abm6570

**Published:** 2022-02-16

**Authors:** Saumya Saurabh, Trisha N. Chong, Camille Bayas, Peter D. Dahlberg, Heather N. Cartwright, W. E. Moerner, Lucy Shapiro

**Affiliations:** 1 Department of Developmental Biology, Stanford University School of Medicine, Stanford, CA, USA.; 2 Department of Chemistry, Stanford University, Stanford, CA, USA.; 3 Department of Plant Biology, Carnegie Institution for Science, Stanford, CA, USA.

## Abstract

Biomolecular condensates formed via liquid-liquid phase separation enable spatial and temporal organization of enzyme activity. Phase separation in many eukaryotic condensates has been shown to be responsive to intracellular adenosine triphosphate (ATP) levels, although the consequences of these mechanisms for enzymes sequestered within the condensates are unknown. Here, we show that ATP depletion promotes phase separation in bacterial condensates composed of intrinsically disordered proteins. Enhanced phase separation promotes the sequestration and activity of a client kinase enabling robust signaling and maintenance of viability under the stress posed by nutrient scarcity. We propose that a diverse repertoire of condensates can serve as control knobs to tune enzyme sequestration and reactivity in response to the metabolic state of bacterial cells.

## Teaser

Bacterial condensates boost enzyme activity under nutrient depletion.

## INTRODUCTION

All cells translate metabolic cues into molecular signals for successful proliferation in dynamic environments. Biomolecular condensates formed by liquid-liquid phase separation (LLPS) of multivalent proteins enable cells to regulate biochemical signals in response to their environment (*1*, *2*). A subset of condensates has been shown to be solubilized at high adenosine triphosphate (ATP) concentrations (*3*) by a mechanism (*4*) that is independent of ATP’s role as a substrate for active processes. Yet, it remains unknown whether modulation of LLPS by ATP has functional relevance for ATP-consuming enzymes compartmentalized within condensates. Bacteria that survive over a broad range of conditions serve as simple yet powerful systems to test the role of ATP depletion on the activity of compartmentalized ATP-consuming enzymes. Here, we studied the function of LLPS in a system of polar condensates that exhibit distinct topologies and physicochemical properties in *Caulobacter crescentus*, an oligotrophic bacterium (*5*). We demonstrate that the activity of a regulatory kinase sequestered to a polar condensate responds to the emergent ATP-dependent physical parameters of the condensate. We show that ATP depletion promotes LLPS, enforces kinase compartmentalization, and sustains kinase activity under nutrient scarcity. Therefore, co-option of the dual roles of ATP as a substrate and as a modulator of LLPS renders cellular fitness under stress induced by nutrient depletion.

**Fig. 1.**
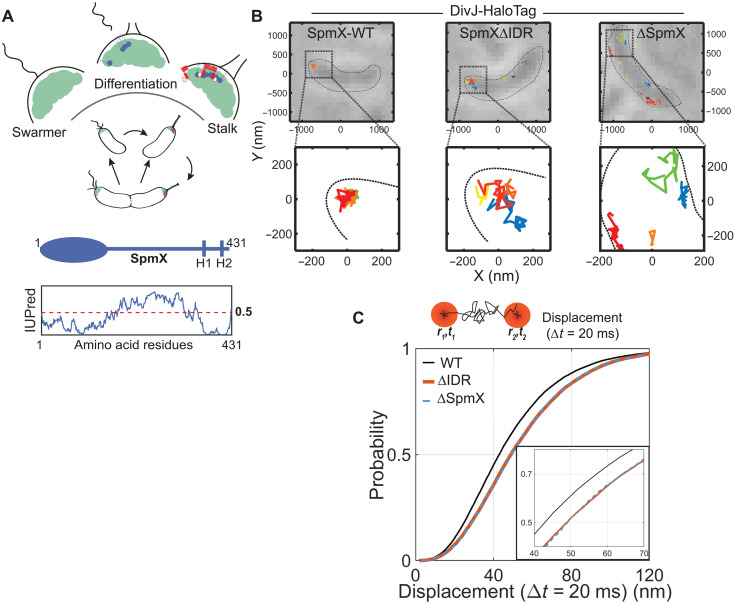
SpmX-IDR sequesters the DivJ kinase at the stalked pole microdomain. (**A**) A schematic of the *Caulobacter* cell cycle showing the key steps of cell division. Zoomed-in schematic above shows the pole during differentiation from swarmer to stalked cell. PopZ (green), SpmX (blue), and the DivJ kinase (red). Shown below is the domain organization and disorder prediction for SpmX calculated using IUPred [H1 and H2 are transmembrane (TM) helices]. Asymmetric localization of the disordered protein SpmX and the DivJ kinase at the stalk-bearing pole is critical for cell cycle progression. (**B**) Top: Representative white light images of live *Caulobacter* cells expressing an endogenous DivJ-HaloTag fusion in strains bearing different SpmX perturbations. Representative single DivJ trajectories are overlaid on the white light images. Approximate cell boundary is indicated using black dashed line. Bottom: A zoomed in view of four polar trajectories (for WT-SpmX and SpmXΔIDR) and nonpolar trajectories (for ΔSpmX). Each trajectory is color-coded and shown as a 2D projection. Polar DivJ exhibits tightly localized trajectories in WT cells while more diffuse trajectories in SpmXΔIDR cells. (**C**) CDF of displacements in 20-ms intervals was obtained from DivJ trajectories in WT, SpmXΔIDR, and ΔSpmX cells. Inset shows a zoomed-in view of the graph highlighting the overlapping CDFs for SpmXΔIDR and ΔSpmX (*N* = 974, 443, and 291, polar trajectories for WT, SpmXΔIDR, and ΔSpmX cells, respectively). In the absence of SpmX-IDR, DivJ exhibited faster diffusivity in the polar microdomain.

The free-living bacterium *C. crescentus* (hereafter, *Caulobacter*) thrives in aquatic habitats and divides asymmetrically, yielding a sessile, stalked cell and a motile, swarmer cell during each cell cycle (Fig. 1A). The swarmer cell is incapable of DNA replication and cell division until it differentiates into a stalked cell (*5*). Differentiation is marked by the loss of flagellum and initiation of stalk biogenesis at the pole. Concomitantly, the polar microdomain composed of the intrinsically disordered protein (IDP) PopZ (*6*) exhibits a change in its client protein composition. A newly expressed protein, SpmX, localizes to the stalk-bearing pole through a direct interaction with PopZ (*7*, *8*). SpmX is an integral membrane, IDP (Fig. 1A) (*9*) that localizes the membrane-associated histidine kinase DivJ (*10*) to the stalked pole. The autokinase activity of DivJ is critical for cell division and depends on its stable localization to the stalked pole, thereby establishing SpmX as a key regulator of the stalked cell developmental program (*7*). Cells harboring a deletion of SpmX exhibit slow growth rates and aberrant cell division, phenocopying a DivJ mutant (*7*). Despite the phenotypic relevance, the precise mechanisms of SpmX-dependent DivJ localization and activity are poorly understood.

How is the activity of relevant kinases such as DivJ regulated under low intracellular ATP concentrations? In addition to its role as a substrate for active cellular processes, ATP has been shown to inhibit LLPS in eukaryotic condensates containing disordered domains (*3*). However, it is unclear whether the role of ATP as an inhibitor of LLPS is conserved in bacteria and whether these mechanisms affect ATP-using enzymes sequestered within condensates. The ATP-dependent kinase reaction of DivJ, in the context of its interactions with SpmX and PopZ, provides a system to test the impact of emergent properties of condensates on the activity of an enzyme that uses ATP as a substrate.

Here, we elucidate the molecular basis for DivJ activity under ATP depletion. We show that DivJ compartmentalization is mediated by a disordered domain in SpmX. SpmX and its localization determinant PopZ form condensates through multivalent interactions that are promoted by their intrinsically disordered regions (IDRs). The multivalence of SpmX and PopZ is shown to be emergent properties in that they can be tuned by ATP within a physiological range. The interplay between ATP levels and SpmX LLPS enabled robust modulation of DivJ activity under low ATP concentrations. Together, our findings elucidate a function of ATP-dependent LLPS in bacterial condensates and underscore its relevance for cellular signaling as a function of nutrient availability.

## RESULTS

### SpmX-IDR slows DivJ diffusion at the pole and stimulates DivJ kinase activity

SpmX has been shown to be indispensable for both DivJ polar localization and kinase activity (*7*). In addition to a structured, lysozyme-like domain that interacts with PopZ (*8*), SpmX contains an IDR of ~200 amino acids (Fig. 1A), whose physiological relevance remains unclear. In vitro, the affinity of DivJ for SpmX is lowered in the absence of the SpmX-IDR (*8*). To determine whether SpmX-IDR influences the polar localization of DivJ in vivo, we performed three-dimensional (3D) single-molecule tracking (*11*) of DivJ (Materials and Methods), endogenously fused to HaloTag and labeled using Janelia Fluor 549 (JF549-HaloTag) (*12*) in SpmX wild-type (WT), SpmX∆IDR, or *spmX* deletion (∆SpmX) strains (Fig. 1B). The DivJ-HaloTag construct is controlled by the native *divJ* promoter fully complemented a ∆*divJ* strain. DivJ exhibited tightly localized polar trajectories in WT cells but diffuse polar trajectories in the SpmX∆IDR cells (Fig. 1B and fig. S1A), indicative of a faster polar diffusion of DivJ in the absence of the SpmX-IDR. The absence of the SpmX-IDR resulted in a decrease in DivJ’s polar concentration. While 60% of all the DivJ trajectories were observed at the pole in WT cells, this number dropped to 20% in SpmX∆IDR cells and to 10% in ∆SpmX cells (fig. S1B). Owing to the small polar diffusivities and limited trajectory lengths, DivJ tracks were analyzed using the cumulative distribution function (CDF) of displacements (*13*) in 20-ms time intervals (Fig. 1C). CDF analyses showed that DivJ displacements in the polar microdomain were larger in both ∆SpmX and SpmX∆IDR cells compared to WT cells (Fig. 1C; fig. S1, C and D; and Supplementary Text), indicating that SpmX and, specifically, the SpmX-IDR increase the residence time of DivJ at the pole. Together with previous data, these results establish a modular role of SpmX in which SpmX polar localization is mediated by an interaction between the SpmX lysozyme-like domain and PopZ (*8*), while the interaction between SpmX-IDR and DivJ results in DivJ’s sequestration within the polar microdomain.

**Fig. 2.**
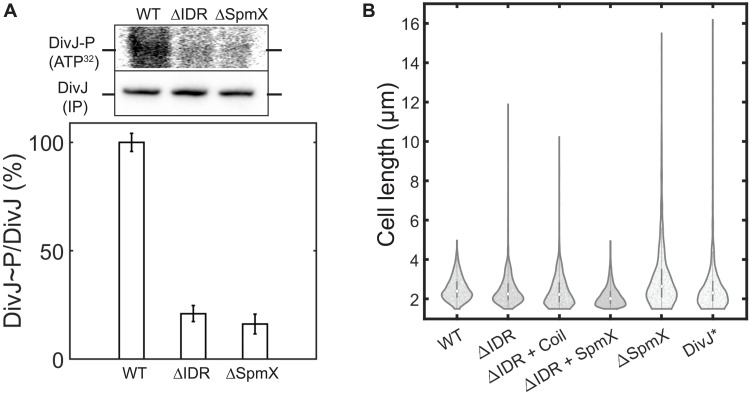
SpmX-IDR stimulates the DivJ kinase. (**A**) In vivo phosphorylation measurement of DivJ in SpmX perturbations. DivJ phosphorylation levels in the bar plot are expressed as a ratio of DivJ~P {intensity in the top gel [ATP(γ − ^**32**^P)]} to DivJ concentration (immunoprecipitation using an antibody against DivJ-HaloTag) and are normalized to the ratio observed in WT cells. ΔIDR denotes SpmXΔIDR. Error bars denote SEM from three biological replicates. SpmX-IDR stimulates DivJ activity in vivo. (**B**) Distribution of lengths for *Caulobacter* cells grown in M2 minimal media supplemented with 0.2 mM glucose for 4 hours, followed by phase contrast microscopy. The white dot on dark gray bars within violin plots denotes the median value of the distribution. ΔIDR denotes SpmXΔIDR; coil denotes a strain endogenously expressing the CoilZ fusion of SpmXΔIDR and a CoilY fusion of DivJ; ΔIDR + SpmX denotes the SpmXΔIDR strain with a mild expression of WT SpmX; DivJ* denotes DivJ(H338A) catalytic mutant. A total of 2000 to 7000 cells were analyzed in each case from three biological replicates. SpmXΔIDR cells mimic a DivJ mutant phenotype under glucose depletion.

Cells lacking SpmX exhibit a marked decrease in DivJ phosphorylation and compromised viability (*7*). To determine whether polar sequestration of DivJ via SpmX-IDR regulates DivJ kinase activity, we measured DivJ phosphorylation levels in vivo in WT, ΔSpmX, and SpmXΔIDR strains using radiolabeled ATP and immunoprecipitation (Fig. 2A and Materials and Methods). Compared to WT cells, SpmXΔIDR strains exhibited an 80% reduction in the levels of phosphorylated DivJ, which was comparable to the levels observed in the *spmX* deletion strain (Fig. 2A). The dependence of DivJ kinase activity on the SpmX-IDR could be a result of a direct conformational regulation of its catalytic domains by the SpmX-IDR or DivJ sequestration within the polar microdomain. Therefore, we tested whether the autokinase activity of DivJ was enhanced by sequestration in its physiological topology in vitro. The cytoplasmic domains of DivJ [DivJ(∆TM)] were tethered to liposomes via an N-terminal His_6_-Tag (Materials and Methods) (*14*). In this assay, we observed a density-dependent increase in DivJ kinase activity, suggesting that DivJ sequestration can enhance its kinase activity in vitro (fig. S1, E and F). While an allosteric regulation of DivJ activity via SpmX-IDR cannot be ruled out, it is plausible that polar sequestration of DivJ via the SpmX-IDR enhances DivJ kinase activity through the effects of increased local concentration that contribute to cooperativity in substrate binding and turnover.

Cells with disrupted DivJ localization or inhibited kinase activity via mutations to DivJ are filamentous due to disruption of cell division (*7*, *15*). Accordingly, we expected SpmX∆IDR cells to be filamentous and to phenocopy a SpmX deletion or a DivJ kinase mutant (fig. S1G). However, the filamentous cell phenotype in SpmX∆IDR cells was observed only under glucose limitation and in stationary phase. Accordingly, we used the distribution of cell lengths for strains grown in low glucose media as a robust phenotypic readout to elucidate the role of SpmX-IDR in DivJ regulation in vivo. While less than 1% WT cells were filamentous (>4 μm), ∆SpmX, SpmX∆IDR, and DivJ kinase mutants exhibited an increased number of cells longer than 4 μm under low glucose (Fig. 2B). The filamentous cell phenotype could be rescued by low expression of WT SpmX in SpmX∆IDR strains (Fig. 2B and fig. S1H). To distinguish between the effect of SpmX-IDR–mediated polar sequestration from other regulatory mechanisms, we constructed a strain where DivJ polar recruitment was independent of the SpmX-IDR. We used cognate coiled domains with a strong binding affinity for this purpose (*16*). A strain with SpmX∆IDR fused to CoilY and DivJ fused to CoilZ exhibited WT-like distribution of DivJ polar localization (Materials and Methods and fig. S1I). However, DivJ localization via a structured coil domain could not rescue the filamentous cell phenotype (Fig. 2B), suggesting that SpmX-IDR sequesters and stimulates DivJ in response to glucose depletion.

Together, the data show that SpmX-IDR is critical for sequestering DivJ at the pole, thereby enhancing its cooperativity and kinase activity. In addition, the SpmX-IDR–mediated cell division phenotype could not be rescued by artificial polar sequestration of DivJ via structured domains, suggesting an allosteric regulatory mechanism that is critical under glucose depletion. The observation of glucose-dependent regulation of DivJ activity suggested that SpmX exhibited an environmental response, which is often observed in condensates containing IDRs (*2*). Accordingly, we tested whether SpmX and PopZ underwent LLPS and quantified the effects of various solvents (including ATP) on their condensate properties.

### SpmX and PopZ form interacting polar condensates via LLPS

**Fig. 3.**
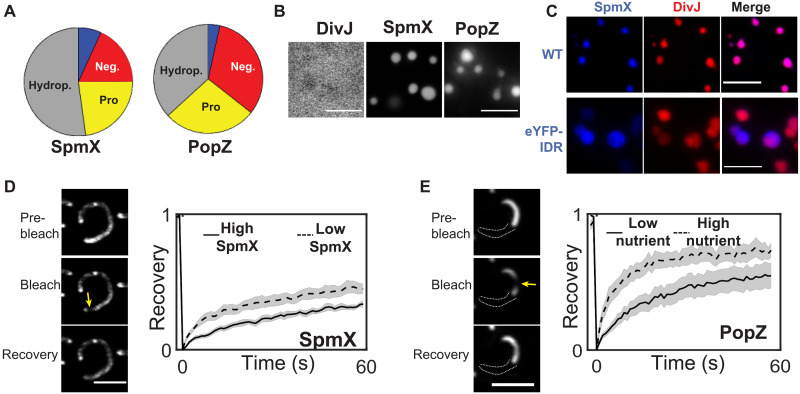
SpmX and PopZ form condensates via LLPS. (**A**) Pie charts depicting the percentage of hydrophobic (gray), prolines (yellow), acidic (red), and basic (blue) residues in SpmX and PopZ IDRs. (**B**) Representative fluorescence images of purified DivJ (ΔTM, 1% labeled with Atto488), SpmX (ΔTM, 5% labeled with Cy3), and PopZ (1% labeled with Atto488, heated at 95°C for 8 min) reconstituted in a physiological buffer [50 mM Hepes-KOH (pH 7.4) and 0.1 M KCl] at 5 μM concentration. DivJ exhibited a diffuse signal, while SpmX and PopZ formed round condensates. (**C**) Fluorescence micrographs showing 5 μM DivJ (ΔTM, 1% labeled with Atto488) incubated with 5 μM SpmX (ΔTM, 5% labeled with Cy3) (top) or 5 μM DivJ (ΔTM, 1% labeled with Cy3) incubated with 5 μM eYFP-tagged SpmX-IDR (bottom) in a physiological buffer for 30 min. DivJ is enriched within SpmX and SpmX-IDR condensates. (**D**) Internal rearrangement of SpmX-eYFP clusters in live *Caulobacter* cells (Δ*popZ*) assayed by fluorescence recovery after photobleaching (FRAP). Yellow arrow in adjacent image shows the bleached spot in the first frame after photobleaching. Recovery plots are shown for low (0.03 % xylose, dashed line, *N* = 11 cells) or high induction (0.3% xylose, solid line, *N* = 12 cells) of SpmX. Gray color represents the SEM. SpmX clusters exhibited concentration-dependent internal dynamics in vivo. (**E**) Internal rearrangement of eYFP-PopZ condensates in live *Caulobacter* cells grown in rich or minimal media assayed by FRAP. Yellow arrow in adjacent image shows the bleached spot. White dashed line denotes an approximate cell boundary. Recovery plots are shown for rich media (blue, *N* = 11 cells) and minimal media (black, *N* = 11 cells). Gray color represents the SEM. PopZ exhibits nutrient-dependent internal rearrangements. Scale bars [(B) through (E)], 5 μm.

The PopZ microdomain has been shown to selectively sequester client proteins at the cell poles (*17*). SpmX is one such client protein that localizes to the stalked pole during differentiation. Both SpmX and PopZ have IDR sequences rich in proline and acidic residues (Fig. 3A). Numerous IDR sequences have been shown to exhibit multivalent interactions that enable formation of biomolecular condensates via phase separation and gelation (*18*, *19*). Accordingly, we determined whether SpmX and PopZ could form condensates under physiological conditions in vitro. Purified PopZ and SpmX exhibited a concentration-dependent increase in turbidity, suggesting the presence of protein assemblies (fig. S2 and Materials and Methods). Microscopic observation of sparsely labeled proteins showed that while DivJ remained soluble in a physiological buffer, SpmX and PopZ formed spherical condensates under similar conditions (Fig. 3B and Materials and Methods). Microscopic observation of purified protein domains revealed that the IDRs of SpmX (AA 156 to 355) and PopZ (AA 1 to 102) were necessary and sufficient for condensate formation in vitro (fig. S2B). Sparsely labeled DivJ partitioned into and was enriched in condensates formed via SpmX or enhanced yellow fluorescent protein (eYFP) fused to SpmX-IDR (Fig. 3C, movies S1 and S2, and Supplementary Text), suggesting that SpmX-IDR can directly interact with DivJ in vitro and supporting the observation of SpmX-IDR–dependent DivJ diffusion in vivo (Fig. 1, B and C).

In vitro, SpmX condensates exhibited spherical morphologies in solution (viewed away from the glass) and relaxed on the aminosilane-treated glass surface. SpmX condensates exhibited moderate internal dynamics just after relaxing on the glass and subsequently underwent rapid gelation, as measured via fluorescence recovery after photobleaching (FRAP) (fig. S2C and Materials and Methods). Rapid fusion events between SpmX condensates were observed within the first minute of condensates fusing on glass, while condensate growth by ripening was observed over longer time scales (fig. S2D). These data suggest that SpmX self-association is enhanced after fusing to glass, further promoting LLPS over time. In vivo, we found that overexpression of SpmX-eYFP in cells lacking PopZ led to the formation of SpmX clusters with high fluorescence intensity in a concentration-dependent manner (fig. S2E). In accord with in vitro measurements, SpmX clusters exhibited concentration-dependent internal rearrangements in vivo, suggestive of increased self-association (Fig. 3D). Notably, fluorescent clusters were not observed upon overexpressing the transmembrane (TM) helices of SpmX fused to eYFP, implicating the cytoplasmic domain in cluster formation (fig. S2F). In vitro, SpmX exhibited a protein and salt concentration–dependent phase behavior. At protein concentrations above 5 μM (0.1 to 0.5 M KCl), condensates deviated from the spherical shape and wetted the glass surface more, marking a region of instability on the phase diagram or gelation suppressing phase separation (fig. S2G) (*1*).

Unlike SpmX, PopZ condensates reconstituted directly from purified stocks deviated from a spherical morphology in vitro (fig. S2H; 23°C sample). 3D confocal imaging revealed the presence of condensates that exhibited a “beads on a string” morphology (fig. S2H and movies S3 and S4), reminiscent of dynamically arrested, poly-arginine–RNA condensates that could be relaxed using thermal energy (*20*). Upon heating at 95°C for 8 min, PopZ condensates transitioned from the beads on a string morphology to a spherical morphology (Fig. 3B, fig. S2H, and Materials and Methods). These observations suggest that PopZ condensates exhibit a lower critical solution temperature behavior (*21*), whereby higher temperature promotes multivalent protein interactions that result in a collapsed, spherical state. Both spherical and aspherical condensates of PopZ exhibited distinct internal rearrangements (fig. S2I) and time-dependent behavior (fig. S2J and Supplementary Text). Overexpression of mCherry-PopZ in *Caulobacter* cells led to an increase in the size of the PopZ microdomain extending from the pole. This observation is in line with the space filling nature of PopZ (*11*) and allowed for the measurement of internal dynamics within PopZ microdomain in vivo. Under the conditions tested, PopZ did not exhibit a concentration-dependent diffusivity. However, for comparable levels of protein expression, we observed a substantial difference in PopZ internal dynamics between cells transferred to high versus low nutrient containing media after protein expression, suggesting a role of metabolites in PopZ diffusion (Fig. 3E). In vitro, the phase behavior of PopZ condensates was a function of protein and salt concentration (fig. S2K). Together, the data show that both SpmX and its localization determinant PopZ use multivalent interactions driven by their IDRs to form condensates that are sensitive to protein concentration and fluctuations in nutrients, temperature, and salt. Observations of context-dependent multivalency in live cells suggest that both SpmX and PopZ form condensates in vivo.

**Fig. 4.**
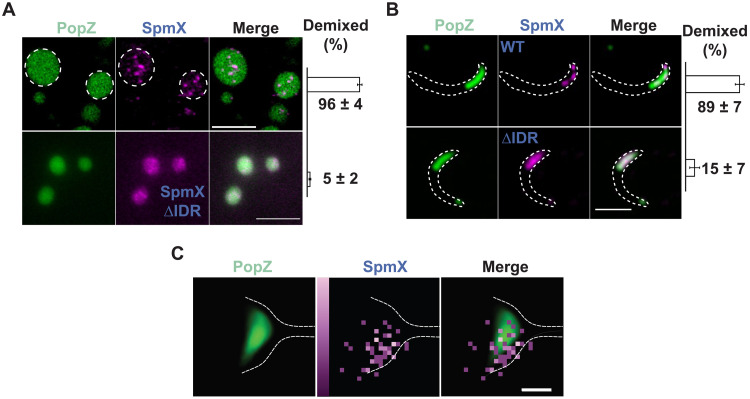
SpmX and PopZ form coexisting yet demixed condensates. (**A**) Representative superresolution images of 5 μM PopZ (1% Atto488 labeled, heated at 95°C for 8 min) incubated with 500 nM (top) SpmX (ΔTM, 5% labeled with Cy3) or (bottom) SpmXΔIDR (ΔTM, 5% labeled with Cy3) proteins in a physiological buffer for 30 min. Adjacent bar plot indicates the percentage of PopZ condensates that exhibited more than one SpmX cluster, serving as a proxy for demixing (*N* ~ 400 condensates per sample). Error bars denote the SD from three biological replicates. SpmX condensate demixing within PopZ condensates is driven by the SpmX-IDR in vitro. Scale bars, 5 μm. (**B**) Superresolution images of cells expressing mCherry-PopZ on a high-copy plasmid and endogenously tagged SpmX-dL5 (top) or SpmXΔIDR-dL5 (bottom). WT SpmX formed multiple clusters associated with the PopZ microdomain, while SpmXΔIDR remained uniformly distributed within PopZ microdomain. Bar plot on the right indicates the percentage of cells that exhibited more than one SpmX cluster within the PopZ microdomain (*N* ~ 230 cells per sample). Error bars denote the SD calculated from three biological replicates. SpmX clusters are demixed within the PopZ microdomain in vivo. Scale bar, 5 μm. (**C**) Left: Manually annotated ribosome excluded region representing the PopZ microdomain from cryogenic electron tomography of four cells. White dashed line denotes an approximate cell boundary. Middle: Localizations of SpmX-PAmKate fusion from correlative cryogenic single-molecule imaging pooled from the same four aligned stalked cells (pixel size, ~14 nm). Color bar to the left of SpmX image denotes the number of localizations binned into each pixel [range 1 (dark) to 3 (light) localizations]. Right: In the overlaid image, SpmX localizations exhibit a nonuniform distribution on one side of the PopZ microdomain, suggestive of demixing. Scale bar, 100 nm.

Next, we investigated the relative topology and mixing behavior of SpmX and PopZ, as they could influence DivJ localization. Superresolution microscopy of samples containing purified PopZ (heated at 95°C for 8 min) and SpmX at a physiological protein ratio exhibited SpmX clusters within 96% of PopZ condensates (Fig. 4A, top, and Materials and Methods). However, PopZ condensates incubated with SpmX∆IDR exhibited a uniformly distributed SpmX∆IDR signal (Fig. 4A, bottom). Cells overexpressing PopZ and harboring a dL5 fusion of SpmX (*22*) as the sole copy expressed at endogenous levels exhibited multiple SpmX polar clusters in 89% of WT cells (Fig. 4B, top) and in 15% of SpmX∆IDR cells (Fig. 4B, bottom). These data suggest that the SpmX-IDR is necessary for driving SpmX self-association and condensate formation within PopZ condensates. In situ imaging of a PAmKate fusion of SpmX in *Caulobacter* cells at cryogenic temperatures (Materials and Methods) followed by overlaying the signal on the annotated ribosome-excluded region (*23*), a proxy for the location of PopZ (*6*), revealed SpmX localizations clustered on one side of the PopZ microdomain (Fig. 4C). While the in situ images are from a small number of cells owing to a limited throughput of correlative imaging, the superior precision (~5 to 10 nm) in SpmX localizations supports the notion that SpmX and PopZ coexist as two interacting yet demixed condensates at the *Caulobacter-*stalked pole.

Cumulatively, the data show that both SpmX and its localization determinant PopZ can form phase-separated condensates via multivalent interactions. Phase separation of PopZ and SpmX is a plausible mechanism enabling polar sequestration of client proteins (*17*), including DivJ, thereby enhancing DivJ’s density-dependent kinase activity. However, SpmX phase separation alone cannot explain the glucose-dependent modulation of DivJ activity by SpmX-IDR (Fig. 2B). We hypothesized that the SpmX condensate could be sensitive to a glucose-dependent metabolite. Since ATP is a key product of glucose metabolism and has been shown to disaggregate eukaryotic condensates at high concentrations in vitro (*3*), we next focused on the role of ATP on SpmX and PopZ condensate formation.

### Solutes tune condensation in a protein-specific manner

Biomolecular condensates that contain disordered proteins can regulate their multivalency by interacting with ligands in their environment (*24*). Therefore, studying the phase behavior of condensates in the context of biologically relevant ligands can elucidate the nature of multivalent interactions. While 1,6-hexanediol (1,6-HD) has been widely used to differentiate between material properties of condensates (*25*), neither is its mechanism of action understood nor produced in cells. More biologically relevant ligands such as ATP and lipoic acid (LA) have been reported to dissolve eukaryotic protein condensates (*3*, *26*). Critically, ATP-mediated dissolution of some condensates has been shown to result from specific ionic interactions mediated by ATP’s negatively charged triphosphate moiety (*4*). Accordingly, we sought to characterize SpmX and PopZ LLPS in the presence of ATP, LA, and 1,6-HD.

**Fig. 5.**
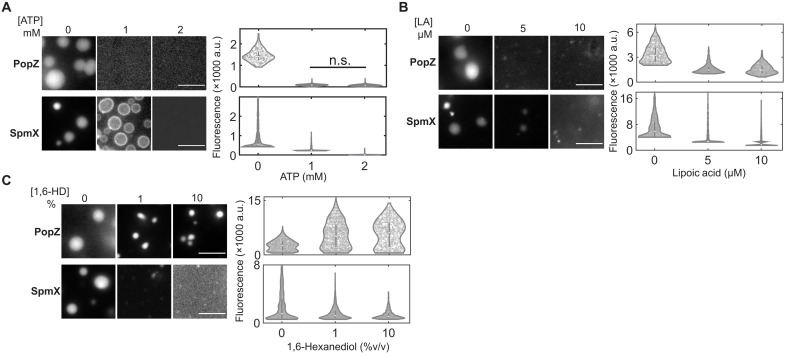
Solutes tune LLPS in a protein-specific manner in vitro. (**A**) Fluorescence micrographs showing 5 μM PopZ (top, 1% Atto488 labeled) or 5 μM SpmX (bottom, ΔTM, 5% Cy3 labeled) incubated with indicated ATP concentrations for 1 hour in a physiological buffer supplemented with 5 mM MgCl_2_. Violin plots (right) show the distribution of the difference in fluorescence signal within the condensate and that outside the condensate, divided by the average fluorescence outside the condensate from a field of view (*N* ~ 600 to 1100 condensates for each nondissolving condition across three biological replicates). ATP can dissolve PopZ and SpmX condensates in vitro. (**B**) Fluorescence micrographs showing 5 μM PopZ (top, 1% Atto488 labeled) or 5 μM SpmX (bottom, ΔTM, 5% Cy3 labeled) incubated with indicated LA concentrations for 30 min in a physiological buffer. Violin plots on the right show the distribution of the fluorescence signal measured as in (A) (*N* ~ 700 to 1100 condensates across three biological replicates). LA can dissolve PopZ and SpmX condensates in vitro. (**C**) Fluorescence micrographs showing 5 μM PopZ (top, 1% Atto488 labeled) or 5 μM SpmX (bottom, 5% Cy3 labeled) incubated with indicated 1,6-HD concentrations for 30 min in a physiological buffer. Violin plots on the right show the distribution of the fluorescence signal measured as in (A) (*N* ~ 600 to 1500 condensates across three biological replicates). Differences between distributions in (A) to (C) are statistically significant (*P* < 0.0005) based on a two-sample *t* test unless denoted by n.s. (not significant). 1,6-HD promotes PopZ condensation while dissolving SpmX condensates in vitro. a.u., arbitrary units. Scale bars, 5 μm.

Microscopic observation of sparsely labeled PopZ and SpmX samples incubated with increasing ATP concentrations showed that both PopZ and SpmX condensates dissolved in the presence of 1 and 2 mM ATP, respectively (Fig. 5A and Materials and Methods). Notably, the dissolution concentrations of ATP for SpmX and PopZ were 5- to 10-fold lower than those observed for eukaryotic condensates (>5 mM) (*3*) but spanned the physiological range of intracellular ATP for bacteria (*27*). In the presence of 1 mM ATP, SpmX formed metastable, hollow condensates that rapidly fused and dissolved (movie S5). Sparsely labeled SpmX and PopZ condensates dissolved upon LA treatment, with PopZ dissolution achieved at lower LA concentrations compared to SpmX (Fig. 5B). While LA is a cofactor for oxidative metabolism in oligotrophic bacteria (*28*), it is bound to proteins and not available freely in cells. Thus, cellular concentrations of LA may not be sufficient for condensate dissolution in vivo. Next, we tested the effect of the aliphatic alcohol 1,6-HD on SpmX and PopZ condensates and found that PopZ condensation was promoted in a 1,6-HD concentration–dependent manner (Fig. 5C), while SpmX condensation was inhibited under the same conditions. PopZ LLPS is likely to be promoted by 1,6-HD through the hydrogen bond acceptor residues (proline, aspartic acid, and glutamic acid) present in its IDR (AA 1 to 102) and a continuous stretch of hydrophobic residues (AA 106 to 127) (fig. S2B, domain diagram).

**Fig. 6.**
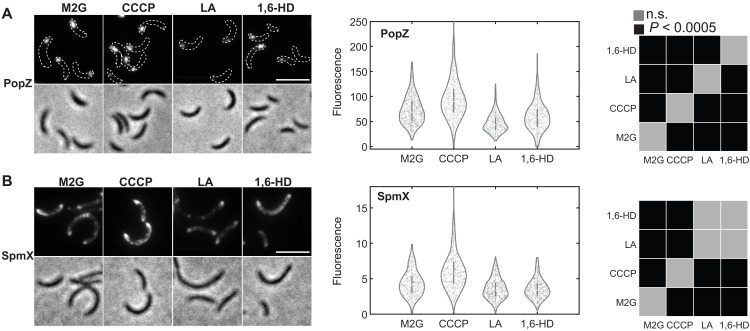
Solutes tune LLPS in a protein-specific manner in vivo. (**A**) Fluorescence and phase contrast micrographs of *Caulobacter* cells overexpressing eYFP-labeled PopZ, untreated (M2G) or treated with 100 μM CCCP (10 min), 5 μM LA, or 5% (v/v) 1,6-HD (30 min, each). Middle panel shows violin plots with distribution of the ratio of localized to diffuse fluorescence within the cell (*N* ~ 900 cells per condition). Right panel shows pairwise statistical significance (*P* values) calculated for each distribution (normal for all cases, lognormal for LA) using a two sample *t* test. In vivo, PopZ multivalency was promoted under ATP depletion and inhibited by LA treatment. 1,6-HD–treated cells showed minimal difference from untreated cells. (**B**) Fluorescence and phase contrast micrographs of *Caulobacter* cells harboring a *popZ* deletion and overexpressing eYFP-labeled SpmX, untreated (M2G) or treated with 100 μM CCCP (10 min), 5 μM LA, or 5% (v/v) 1,6-HD (30 min, each). Middle panel shows the distribution of the ratio of localized to diffuse fluorescence within the cell (*N* ~ 700 cells per condition). Right panel shows pairwise statistical significance (*P* values) calculated for each distribution (normal for all cases, lognormal for LA) using a two-sample *t* test. SpmX multivalency was promoted under ATP depletion and inhibited by LA and 1,6-HD treatment in vivo. Scale bars, 5 μm.

The intracellular environment may affect fluctuations in ligand concentrations, necessitating investigation of the observed in vitro stimulus responses in living cells. Accordingly, we tested the effect of rapid ATP depletion by the ionophore carbonyl cyanide *m*-chlorophenyl hydrazone (CCCP) (fig. S3A) or exposure to LA and 1,6-HD on PopZ and SpmX assemblies in live *Caulobacter* cells. To clearly differentiate between cluster intensities, we used mild overexpression of fluorescently tagged PopZ and SpmX for these experiments. Cells overexpressing eYFP-PopZ (Fig. 6A) or SpmX-eYFP (Fig. 6B) were treated with 100 μM CCCP (10 min), 5 μM LA, or 5% 1,6-HD (30 min, each) (Materials and Methods). Using the ratio of fluorescence from protein clusters to cellular background as a proxy for degree of condensation, we observed that depletion of ATP by CCCP addition promoted condensation for both PopZ and SpmX in vivo. While LA inhibited condensation for both proteins (Fig. 6, A and B), 1,6-HD treatment depleted only SpmX condensation. The similarity between the solute-dependent condensation of SpmX and PopZ, in vitro and in vivo experiments, further supports the notion that clusters observed in live cells are phase-separated condensates.

As a negative control, overexpression of DivJ-eYFP from a high copy plasmid in a *spmX* deletion strain led to the formation of multiple DivJ clusters throughout the cell (fig. S3B). However, unlike SpmX or PopZ condensates, DivJ-eYFP clusters exhibited a mild reduction in signal under all solute treatments. As another control, we measured the effect of the ligands on the self-assembly of the structured intermediate filament protein Crescentin (CreS), which forms an extended filament on the ventral (concave) side of *Caulobacter* cells (*29*). Similar to DivJ, CreS-eYFP fibers exhibited no change in fluorescence upon CCCP treatment, but they exhibited a slight increase in fluorescence upon LA or 1,6-HD treatment (fig. S3C). Last, as a positive control, we treated cells expressing ribonuclease E (RNase E)–eYFP, a key component of the *Caulobacter* RNA degradosome organelle (bacterial ribonucleoprotein bodies) (*30*) with CCCP, LA, and 1,6-HD. RNase E–eYFP condensation was promoted by ATP depletion and 1,6-HD treatment while being inhibited under LA treatment, compared to untreated cells (fig. S3D). To assess the effect of chemical perturbations on cell health, we used a cell impermeant fluorescent dye as a proxy for membrane leakage (Materials and Methods). While LA- and CCCP-treated samples showed a similar number of cells with compromised membranes compared to untreated samples (~1 to 2%), 1,6-HD–treated samples exhibited ~8% cells with membrane leakage (fig. S3E). The effects of 1,6-HD on membrane integrity shown here and enzyme activity reported previously (*31*) emphasize the pleiotropic consequences of the compound in vivo.

Cumulatively, the data show that phase separation within three different *Caulobacter* condensates is promoted at low ATP levels and inhibited by LA, whereas structured protein assemblies (CreS) and protein-rich aggregates (DivJ overexpression in the absence of SpmX) do not exhibit such a behavior. While 1,6-HD inhibits SpmX condensation, it promotes PopZ condensation likely by promoting multivalent interactions via hydrophobic residues and hydrogen bond acceptor moieties. Critically, the data challenge the widely held dogma that molecules such as 1,6-HD generally inhibit LLPS while supporting the notion of protein- and ligand-specific effects on multivalency and cell membrane integrity. The data also underscore that the assessment of liquid or gel-like states using 1,6-HD in vivo should be considered in the metabolic context of the cell. Having provided evidence that ATP modulates SpmX LLPS, we asked whether ATP’s role as a solute of SpmX condensates is relevant for the DivJ kinase regulation.

### ATP-dependent modulation of DivJ activity via SpmX LLPS

Given that DivJ exhibited density-dependent kinase activity (fig. S1E), we asked whether ATP depletion in the cell promoted SpmX condensation and led to enhanced DivJ polar sequestration and kinase activity. Accordingly, we measured whether DivJ exhibited ATP-dependent polar localization in strains bearing WT SpmX. Cells overexpressing SpmX-eYFP and DivJ-mCherry in a *popZ* deletion strain exhibited enhanced DivJ sequestration under CCCP triggered ATP depletion (fig. S4A), a phenotype that was not observed in the absence of SpmX (fig. S3B). Next, we tested whether ATP-dependent DivJ sequestration via SpmX-IDR led to a metabolic control of cell division in stalked cells. We used aberrant cell division as a proxy for DivJ activity as before (Fig. 2B) while growing cells in minimal media with varying glucose concentrations. Cells grown under a fivefold lower glucose concentration for 4 hours exhibited ~18% decrease in ATP levels (fig. S4B), measured using a commercial luciferase-based assay (Materials and Methods).

**Fig. 7.**
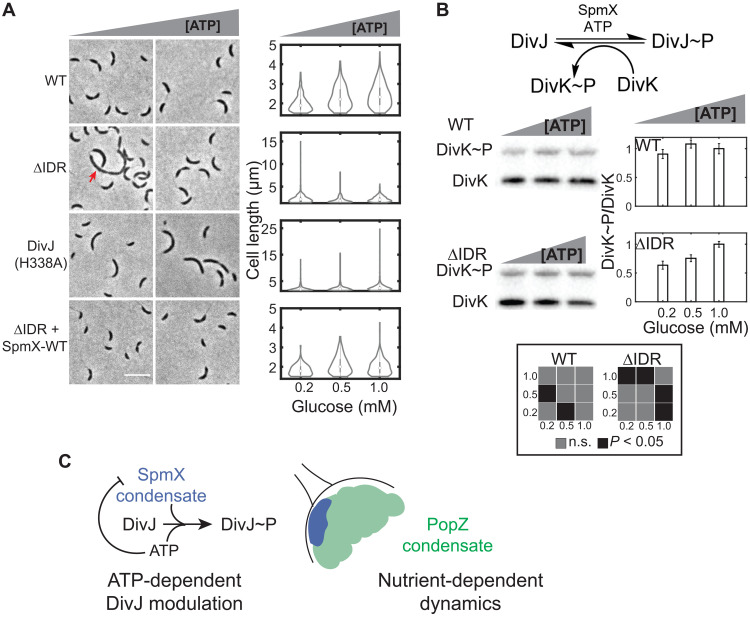
ATP-dependent modulation of DivJ activity via SpmX LLPS. (**A**) Representative phase contrast images of WT cells (top), SpmXΔIDR cells, DivJ(H338A) cells, and SpmXΔIDR cells with mild expression of WT SpmX (bottom), grown in minimal medium with varying glucose concentrations [0.2 mM (left) to 1 mM (right)]. Red arrow in the ΔIDR strain imaged under 0.2 mM glucose denotes an aberrantly dividing cell. Violin plots on the right show the cell length distribution as a function of glucose and intracellular ATP concentrations. Note the differences in the ordinate for WT (1 to 5 μm), SpmXΔIDR (1 to 15 μm), and the DivJ mutant (1 to 25 μm). SpmXΔIDR exhibits an ATP-dependent cell length phenotype, phenocopying WT cells under high ATP, and the DivJ kinase mutant under low ATP concentrations. Mild expression of WT SpmX in cells harboring SpmXΔIDR rescues the division phenotype. Scale bar, 5 μm. (**B**) Top: Schematic showing the DivJ kinase reaction. SpmX-IDR and ATP promote DivJ phosphorylation while DivK dephosphorylates DivJ. Bottom and left: PhosTag SDS–polyacrylamide gel electrophoresis (SDS-PAGE) immunoblots showing DivK phosphorylation in extracts from stalked and predivisional WT (top) or SpmXΔIDR cells (bottom) as a function of ATP levels. DivK was fused to a Flag peptide and detected via an anti-Flag antibody. Bottom and right: Bar plots show the ratio of phosphorylated and unphosphorylated DivK measured from three replicates of the PhosTag assay. Inset shows pairwise statistical significance (*P* values) calculated using a two-sample *t* test. DivK (and, by extension, DivJ) phosphorylation is robust to ATP fluctuations in WT cells but sensitive to ATP levels in SpmXΔIDR cells. (**C**) Model showing the topological relationship between SpmX and PopZ condensates. PopZ and SpmX condensates exhibit nutrient-dependent dynamics. The SpmX condensate renders ATP-dependent feedback on DivJ, resulting in robust modulation of DivJ under glucose depletion.

In the WT strain, the distribution of cell lengths broadened with higher intracellular ATP concentrations (Fig. 7A and Materials and Methods). A DivJ kinase mutant strain exhibited ~10-fold higher number of filamentous cells compared to WT cells. The number of observed filamentous cells in the DivJ kinase mutant was proportional to ATP levels, as observed for length distributions in the WT strain. In stark contrast to WT or DivJ kinase mutant cells, SpmX∆IDR cells grown under high glucose phenocopied WT cells and under low glucose phenocopied the DivJ kinase mutant (Fig. 7A). This phenotype could be rescued by mild expression of a WT copy of *spmX* under the control of a chromosomal xylose promoter in SpmX∆IDR cells (Fig. 7A and fig. S1H). The notion of ATP-dependent DivJ modulation was also supported by cell growth assays, where the rate of increase in cellular biomass was three times slower for SpmX∆IDR cells compared to WT cells under low glucose concentrations (fig. S4C).

To test whether aberrant cell divisions in SpmX∆IDR cells were a result of reduced DivJ kinase activity, we sought to measure the levels of phosphorylated DivJ in lysates from cells using PhosTag gel electrophoresis as a function of decreasing glucose in the growth media (Materials and Methods). Owing to the low copy numbers of DivJ (~12 molecules per pole) (fig. S4D) and the labile nature of phospho-histidine, we were unable to robustly measure DivJ~P levels using PhosTag assay. Therefore, we relied on DivJ’s response regulator DivK (Fig. 7B) as a proxy for DivJ~P levels in stalked and predivisional cells. DivK dephosphorylates DivJ (fig. S4E) thereby forming a relatively stable phospho-aspartate bond allowing a robust measurement via PhosTag. WT and SpmX∆IDR cells expressing an endogenous Flag-tagged copy of DivK were grown under decreasing glucose concentrations. Lysates from these cells were subjected to PhosTag gel electrophoresis, where phosphorylation-dependent protein migration enables separation between phosphorylated and unphosphorylated forms of the same protein. PhosTag gels were immunoblotted, and both forms of Flag-tagged DivK were detected using an antibody against Flag-tag. While the DivK~P/DivK ratio correlated with the decrease in glucose (ATP) for SpmX∆IDR cells, such an ATP dependence was not observed in WT cells (Fig. 7B). These data show that ATP and SpmX-IDR–dependent regulation of cell length are due to changes in DivJ phosphorylation levels and underscore the importance of SpmX-IDR in promoting DivJ activity under ATP depletion. Cumulatively, the data establish SpmX as a membrane-associated condensate that compartmentalizes DivJ and promotes its kinase activity. The sensitivity of SpmX condensation to physiological intracellular ATP levels acts as feedback to enhance DivJ cooperativity and activity, particularly when its substrate ATP is scarce, thereby enabling a robust modulation of the DivJ kinase.

## DISCUSSION

Cellular processes critical for survival and adaptation must be robust across a broad range of nutrient conditions. Unlike growth in laboratory media, bacteria in their natural habitats experience large fluctuations in nutrient concentrations (*32*) and must adapt their biochemistry for survival particularly under nutrient depletion. Yet, mechanisms that connect the metabolic state of the cell to its biochemical signaling are poorly understood. Here, we investigated the mechanisms that impart robustness to the DivJ kinase under ATP limitation in the aquatic bacterium, *C. crescentus*. We demonstrate that DivJ polar localization and kinase activity are promoted by the disordered domain of SpmX. SpmX and its localization determinant PopZ form interacting but demixed condensates through multivalent interactions. Our data show that SpmX-IDR promotes LLPS and stimulates DivJ through enhanced cooperativity and IDR-mediated molecular organization. A similar molecular organizing role in addition to cooperativity has been recently observed in vitro in a eukaryotic condensate system (*33*). Chemical probing of SpmX and PopZ condensates revealed solute- and protein-specific effects on LLPS. While it is not possible to ascertain the specific phase behavior of SpmX and PopZ in living cell due to size constraints, the observation of context-dependent protein partitioning supports the idea that these proteins exist as biomolecular condensates in vivo. Critically, the data show that ATP depletion promoted LLPS in SpmX and PopZ condensates in vivo. The ATP-dependent condensation of SpmX conferred feedback on DivJ sequestration, promoting DivJ kinase activity under low ATP concentrations. Our observations explain the nutrient-dependent dynamics of the PopZ condensate and underscore the relevance of the SpmX condensate in DivJ regulation under low glucose conditions (Fig. 7C).

While certain eukaryotic cells and symbiotic bacteria often thrive in relatively stable environments, free-living bacteria are constantly exposed to chaotic cycles of famine and feast. Many enzymes critical for cell proliferation are present at low copy numbers, which poses an additional challenge for cells surviving under substrate depletion. Condensates formed by multivalent interactions among disordered proteins enable selective sequestration of low copy number enzymes (*17*) and buffer against the noise associated with stochasticity in enzyme activity (*34*). Our results suggest that compartmentalization of a low copy number kinase within an ATP-sensitive condensate is an efficient mechanism to further reduce the biochemical noise under nutrient depletion. Notably, such a mechanism enables an ATP-dependent degree of cooperativity of an enzyme, as opposed to a fixed degree of cooperativity (such as the Hill coefficient), and boosts enzyme activity under substrate limitations. Further, because of faster diffusion of ATP compared to gene expression time scales, condensates sensitive to intracellular ATP concentration range may adapt swiftly and robustly to changing metabolic conditions.

In eukaryotic cells, high levels of intracellular ATP (5 to 10 mM) have been attributed to the maintenance of a soluble proteome. Why are bacterial condensates soluble within a much lower ATP range (0.5 to 1 mM) compared to eukaryotic condensates? It has been observed that the intrinsic disorder content of a proteome is correlated with cellular complexity (*35*). Thus, we posit that the emergent ATP-dependent LLPS via disordered proteins has been preserved through evolution along with a concomitant increase in cellular complexity and energy consumption. This idea is also supported by recent observations of ATP-dependent protein condensation (*36*) and aggregation (*37*) in bacteria. ATP has been shown to affect the diffusion of mesoscopic macromolecular assemblies in bacteria (*38*, *39*). Our work reveals that modulation of LLPS by ATP affects molecular diffusion even within mesoscopic condensates and may be a critical mechanism for optimal functioning of compartments formed by disordered proteins. Thus, we propose that the diverse repertoire of condensates can serve as control knobs to tune reactivity in response to the metabolic state of the cell and promote bacterial fitness and adaptation.

## MATERIALS AND METHODS

### Strain engineering

A list of all strains, plasmids, and primers used in this study is provided in the table S1. For making *Caulobacter* strains, a set of plasmids reported previously (*40*) was used. pTS18 was constructed by inserting eYFP into pAP515 using Kpn I and Nhe I restriction sites by T4 ligation. pTS23 was constructed by SpmX [amino acid (AA) 1 to 162]-eYFP-SpmX (AA 356 to 431) synthesis and insertion into plasmid pMCS-4 using Eco RI and Nhe I restriction sites. pTC276 was constructed by amplification of divK plus 495 base pairs upstream of divK with primers TC540F and TC541R and 800 base pairs downstream of divK with primers TC544F and TC545R from NA1000 genomic DNA. These two DNA fragments along with a gBlock containing the synthesized sequence for Halo-3xFlag with 5′ and 3′ overlapping sequences were inserted into the plasmid pNPTS-138 using restriction sites Spe I and Eco RI by Gibson assembly. pTC8 was constructed by amplification of mCherry with primers TC29F and TC30R and inserted into a pBX-popZ plasmid (*6*) using the Nde I restriction site and Gibson assembly. pSS083 was constructed by inserting a synthesized piece of HaloTag with appropriate overhangs into pAP515 using Kpn I and Nhe I restriction sites by T4 ligation. pSS225 was constructed by inserting a synthesized piece of HaloTag-CoilY with appropriate overhangs into pAP515 using Kpn I and Nhe I restriction sites by T4 ligation. pTS35 was constructed by inserting a synthesized fragment of SpmX (AA 1 to 162)-eYFP-CoilZ-SpmX (AA 356 to 431) into pTS23 using Nhe I and Eco RI restriction sites by T4 ligation. pSS47 was constructed by amplifying PopZ from *Caulobacter* genomic DNA using primer SS013F and adding the Tobacco Etch Virus protease cleavage site and His6 sequence on the reverse primer SS014R. The resulting fragment was inserted into pet28a vector digested using Eco RI restriction site by T4 ligation.

An Amber codon containing SpmX (1 to 355) was synthesized and inserted into a pet28a vector. pSS121 was constructed by amplifying the sequence for SpmX (AA 1 to 355) using primer SS7F and adding the TEV site and His10 on the reverse primer SS8R. The resulting fragment was inserted into pet28a vector digested using Nco I and Bam HI restriction sites. pSS102 was constructed by amplifying PopZ (AA 1 to 102) with TEV and His6 from pSS47 using primer SS15F and SS16R followed by insertion into pet28a vector digested using Eco RI restriction site by T4 ligation. pSS103 was constructed by amplifying PopZ (AA 103 to 177) with TEV and His6 from pSS47 using primers SS17F and SS18R followed by insertion into pet28a vector digested using Eco RI restriction site by T4 ligation. pSS120 was constructed by amplifying SpmX (AA 1 to 155) from pSS43 using primers SS9F and SS10R and inserting the resulting fragment into pet28a vector digested using Nco I and Bam HI restrictions sites. pSS165 was constructed by synthesizing an eYFP sequence fused to SpmX (AA 156 to 355)-TEV-His10 sequence and inserting the resulting fragment into pet28a vector digested using Nco I and Bam HI restrictions enzymes. pSS306 was constructed by amplifying the DivJ coding sequence from pAP515 using primers SS321F and SS322R and inserting the resulting fragment into pVCHYC-2 digested using Nde I and Kpn I restriction enzymes. pSS319 was constructed by amplifying the DivJ-eYFP sequence from pTS18 and inserting the resulting fragment into pBXMCS-4 plasmid digested using Nco I and Kpn I.

TS4 was constructed by transduction of SS087 with ΦCr30 phage carrying pspmX:spmX-dL5 (*22*, *41*) and selected by gentamycin resistance. TS5 was constructed by transduction of ∆*spmX* with phage carrying pdivJ:divJ-HaloTag and selected by chloramphenicol resistance. TNC17 was constructed by electroporating NA1000 cells with plasmid pTC8 and selection for gentamycin resistance. TS15 was constructed by electroporation of ∆*divJ* cells with plasmid pTS18 and selection for chloramphenicol resistance. TS34 was constructed by electroporation of SS087 cells with plasmid pTS23 and selection for gentamycin resistance. SS057 was constructed by electroporating pSS47 in BL21(DE3) cells. SS297 was constructed by electroporating the plasmid pBXyl-SpmX-eYFP in a *popZ* deletion strain. This strain was constructed to observe differences in SpmX partitioning within clusters, which could not be reproducibly measured under native expression conditions, possibly due to the low copy number of SpmX molecules. SS311 was constructed by electroporating pSS306 in SS297. SS235 was constructed by electroporating pTS35 into AP369 cells followed by a second electroporation with the plasmid pSS225. SS324 was constructed by electroporating pSS319 into AP369.

SS294 and SS295 were constructed by electroporating TS4 or TS34 with plasmid pTC276. Following selection for kanamycin resistance, recombination of transgenic cells was allowed to occur overnight in peptone yeast extract (PYE) liquid culture containing no antibiotics. Recombinant lines having lost the *sacB* gene in the pNPTS-138 backbone that confers sucrose sensitivity were selected for on PYE plates containing 3% sucrose. Recombinant lines containing divK-HaloTag-3xFlag were confirmed by polymerase chain reaction (PCR) of lysed cells using primers TC532F and TC533R. The DNA for the TM domain of the protein ArcB (Aerobic respiration control sensor protein, P0AEC3) from *Escherichia coli* was synthesized with a glycine-serine linker (GSx4) with overhangs to complement the xylose locus on one end and the HaloTag locus on the other. The plasmid was then constructed via Gibson assembly into the vector backbone pBXMCS-6 (*42*). The resulting plasmid was electroporated into WT or Δ*spmX* strains and selected with appropriate antibiotics to obtain SS150 and SS159, respectively. The peptide sequence of the TM domain is MKQIRLLAQYYVDLMMKLGLVRFSMLLALALVVLAIVVQMAVTMVLHGQVESIDVIRSIFFGLLITPWAVYFLSVVVEQLEESRQR.

### Recombinant protein purification and labeling

#### Protein expression

All recombinant proteins used in this study were purified using isopropyl-β-d-thiogalactopyranoside (IPTG)–inducible T7 expression vectors. The cytoplasmic portion of SpmX (AA 1 to 355) was expressed in a modified pet28a vector to contain a C-terminal TEV proteolytic site followed by a polyhistidine tag (His-10). The codon encoding for residue 338 was modified from a codon for phenylalanine to an Amber codon. This was done to enable labeling the protein molecule with a single dye using click chemistry. BL21(DE3) cells were cotransformed with the protein-bearing pet28a-SpmX(1-355)-TEV-His10 plasmid and the transfer RNA (tRNA) synthetase and tRNA-expressing plasmid pEvol-pAzF (*43*) for incorporation of *p*-azido-l-phenylalanine into proteins in response to the Amber codon, TAG. pEVOL-pAzF was a gift from P. Schultz (Addgene plasmid no. 31186). BL21 cells containing these plasmids were grown overnight in 2x-yeast triptone (YT) media at 37°C. The next morning, cells were diluted 100-fold in 1 liter of 2x-YT media supplemented with kanamycin (20 μg/ml) and chloramphenicol (30 μg/ml) and grown in 2.8-liter baffled flasks at 37°C until they reached an optical density at 600 nm (OD_600_) of 0.3. At this stage, 238 mg of IPTG, 200 mg of l-arabinose, and 200 mg of *p*-azido-l-phenylalanine (dissolved in 2 ml of 0.1 N NaOH) were added to the flasks. The flasks were then moved to a 30°C incubator where protein induction was carried out for 4 to 5 hours. After induction, the cells were washed using cell wash buffer [50 mM tris (pH 7.4) and 500 mM NaCl] and pelleted in 50-ml conical tubes by spinning at 3700*g* for 15 min. The pellets were saved in a freezer at −80°C until they were ready to be used. SpmX∆IDR (AA 1 to 155), PopZ (AA 1 to 177), PopZ (AA 1 to 102), PopZ (AA 103 to 177), DivJ∆TM (AA 188 to 597), DivK, and DivK (D53N) were all expressed without Amber codons in pet28a vector containing TEV protease and polyhistidine sites. We observed that SpmX (156 to 355) was unstable in *E. coli*. To circumvent this, we expressed SpmX (AA 156 to 355) as a N-terminal fusion to eYFP. The resulting protein eYFP-SpmX-IDR (AA 156 to 355)-TEV-His10 was more stable than the untagged SpmX-IDR. Proteins expressed without Amber codons were purified using the same protocol as above with appropriate antibiotics, except that the cells were grown in LB, and only IPTG was added during the induction step.

#### Protein purification

For purifying proteins from BL21 cells, frozen pellets were thawed on ice in 50-ml conical flasks with prechilled lysis buffer [50 mM Hepes-KOH (pH 7.6), 500 mM KCl, Roche Protease Inhibitors tablet (0.5 tablet/liter of culture), 25 mM imidazole, 200 U benzonase nuclease (0.3 μl/1 liter culture)]. Cells were resuspended in lysis buffer and lysed using sonication. The sonicated sample was centrifuged at 29,000*g* for 45 min in a JS-20 rotor at 4°C. The supernatant was collected and incubated with 2 ml of preequilibrated nickel-nitriloacetic acid (Ni-NTA) agarose slurry (50%) for each liter of spun down cells. The slurry was nutated for 2 hours at 4°C. The slurry was then washed three times with Ni-NTA wash buffer [50 mM Hepes-KOH (pH 7.6), 500 mM KCl, and 25 mM imidazole], and protein was eluted from the slurry by incubating the slurry with elution buffer [50 mM Hepes-KOH (pH 7.6), 500 mM KCl, 250 mM imidazole, and 10% glycerol] for 20 min and collecting the flowthrough. Proteins were then dialyzed twice against dialysis buffer [50 mM Hepes-KOH (pH 7.4), 100 mM KCl, and 10% glycerol]. Dithiothreitol (DTT) was avoided in dialyses where the protein was to be subsequently labeled using a fluorescent dye, as DTT reduced fluorophores and affected multivalency within condensates. Proteins that did not contain an IDR were further purified by size-exclusion chromatography. Proteins were concentrated to appropriate stocks and flash-frozen in liquid nitrogen and frozen at −80°C until needed.

#### Protein labeling

For SpmX labeling with Cy3-DBCO using click chemistry, the dye was reconstituted in the dialysis buffer and incubated with the eluted protein at 20× molar excess overnight at 4°C. In the case of proteins that were labeled using Atto488 *N*-hydroxysuccinimide (NHS) dye, labeling was performed by using 20× molar excess of Atto488-NHS for 2 hours at 4°C. Free dye was removed from the samples by gravity-assisted chromatography on a PD-10 column (Cytiva, 17-0851-01). Proteins were concentrated to appropriate stocks and flash-frozen in liquid nitrogen and frozen at −80°C until needed. Labeled and unlabeled proteins were mixed at the requisite ratio for 1 hour at room temperature before the experiments.

### In vitro reconstitution

#### Imaging chamber preparation

Droplet experiments for SpmX phase separation were performed in glass bottom 384-well plates (MatTek, PBK384G-1.5-C). The wells were treated by flushing them with nitrogen gas followed by the addition of 50 μl of freshly prepared 1 M KOH in MilliQ water. The plate was centrifuged for 10 s at 600*g* to ensure that the liquid has settled down, followed by a 10-min incubation at room temperature with slow shaking. Next, the wells were washed five times with 50 μl of MilliQ water. After the final wash, 20 μl of (3-aminopropyl)triethoxysilane (APTES; Vector Laboratories) was added to each well. The plate was centrifuged for 10 s at 600*g* to ensure that the liquid has settled down. The wells were covered with an aluminum foil and incubated for 1 hour at room temperature with slow shaking. The wells were washed five times with 50 μl of phosphate-buffered saline per wash and five times with 50 μl of water per wash. The wells were dried for 1 to 2 hours in the dark at room temperature followed by sample addition and imaging. In general, we observed a deterioration in the quality of the chemically modified surface after 1 day of the treatment. Therefore, this procedure was performed on the day of the experiments.

#### Protein treatment

Poly-histidine tags were cleaved from proteins by treating them with TEV protease (Sigma-Aldrich) overnight at 4°C based on the manufacturer’s guidelines. TEV-proteolyzed unlabeled proteins were mixed with TEV-proteolyzed dye-labeled proteins at the indicated mole percent, which was 1% for PopZ-Atto488, 5% for SpmX-Cy3 (or SpmX∆IDR-Cy3), 1% for DivJ-Atto488, and 1% for DivJ-Cy3. eYFP fusion of SpmX-IDR was used without any additional mixing steps. PopZ samples were prepared by mixing labeled and unlabeled proteins to obtain a stock, followed by heating the samples in PCR tubes at 98°C for 8 min.

#### Condensate imaging

Microscopic observation of condensates was performed by diluting the TEV-proteolyzed proteins to 5 μM in a physiological buffer [50 mM Hepes-KOH (pH 7.4) and 100 mM KCl]. Where needed, proteins were mixed at the respective concentrations in the physiological buffer. Protein solutions were then incubated in PCR tubes at room temperature for 30 min followed by addition of 30-μl samples into the APTES-treated chambers in glass bottom 384-well plates. The plate was then mounted on the microscope stage. Condensates could be observed in solution, away from the glass followed by settling to the bottom of the chamber within 10 to 20 min. The eYFP-fused SpmX-IDR protein was an exception to this observation. eYFP-IDR condensates collapsed upon touching the APTES-treated glass surface. Other surface modifications were tried for eYFP-IDR (KOH, poly-l-lysine, bovine serum albumin, poly-l-lysine–PEG, or untreated glass with or without Argon plasma etching), with no success. As a result of this surface-induced instability, eYFP-IDR condensates (with or without DivJ-Cy3) were imaged while in motion before they fused on the glass surface.

#### Salt-dependent condensate phase diagram imaging

For imaging condensates without any salt, an aliquot of concentrated protein was dialyzed against a buffer lacking any KCl [50 mM Hepes-KOH (pH 7.4) and 10% glycerol] using minidialysis cassettes [Slide-A-Lyzer MINI Dialysis Device, 3.5 K molecular weight cutoff (MWCO) and 0.1 ml] for 1 hour at room temperature. The dialyzed and desalted protein was then taken up at appropriate concentrations in the imaging buffer [50 mM Hepes-KOH (pH 7.4) and 10% glycerol]. For 0.1 M KCl, the purified protein was diluted to appropriate concentrations in imaging buffer containing 0.1 M KCl, and for higher KCl concentrations (0.2 to 0.5 M), additional KCl dissolved in the imaging buffer was supplemented into the reactions.

#### Condensate imaging under chemical perturbations

For experiments requiring ATP addition, 5 μM protein samples were prepared as above and incubated at room temperature for 30 min before addition of the requisite concentrations of ATP in imaging buffer [50 mM Hepes-KOH (pH 7.4), 100 mM KCl, and 5 mM MgCl_2_]. ATP stocks were prepared in water at 500 mM concentration, and pH was adjusted to 7.4 by adding 1 N NaOH. Subsequent dilutions of the 500 mM ATP stock were made in the imaging buffer [50 mM Hepes-KOH (pH 7.4), 100 mM KCl, and 5 mM MgCl_2_]. Addition of the highest concentration of ATP led to a change in the ionic strength of the solution from 115 to 123 (7%). Using a control buffer with matched ionic strength [50 mM Hepes-KOH (pH 7.4), 108 mM KCl, and 5 mM MgCl_2_], we validated that the observed effects of ATP on condensates were not due to changes in ionic strength. Similar experiments were performed using adenosine diphosphate (ADP) and Adenosine (b,γ-imido)triphosphate (AMP-PNP). SpmX and PopZ condensates dissolved in the presence of both ADP and AMP-PNP at concentrations ranging from 0.5 to 2 mM. LA stocks were made in dimethyl sulfoxide (DMSO) at 500 μM, and dilutions of the stock were made in imaging buffer [50 mM Hepes-KOH (pH 7.4) and 100 mM KCl]. As a control for experiment requiring LA addition, an equal volume of DMSO was added to the protein samples. 1,6-HD was purchased as a 99% aqueous solution and was added at desired concentrations to proteins. In this case, an equivalent volume of water was added to the untreated samples as a control.

#### Turbidity measurement using absorbance

To assess turbidity in protein samples, absorbance at 600 nm was measured in 384-well plate format using a Varioskan LUX multimode microplate reader (Thermo Fisher Scientific). Unlabeled SpmX or PopZ was reconstituted in a physiological buffer [50 mM Hepes-KOH (pH 7.4) and 0.1 M KCl] at increasing concentrations. Protein samples were added to a 384-well polystyrene plate and incubated for 30 min at room temperature before measuring absorbance on a plate reader. As a control, purified DivJ and HaloTag were used at the same concentration range and did not exhibit any concentration-dependent turbidity. In all cases, the buffer-only background was subtracted from the absorbance values.

### In vitro DivJ kinase assays

#### Liposomes

DivJ phosphorylation assay on liposomes was performed on nitrocellulose blots as described previously (*14*, *44*). Small unilamellar vesicles (SUVs) were prepared by dissolving polar phospholipids (Avanti Polar Lipids, Alabaster, AL) in chloroform at a 9:1 molar ratio {nine parts 1,2-dioleoyl-sn-glycero-3-phospho-(1′-rac-glycerol; sodium salt; di-oleoyl-phosphatidyl glycerol: product 840475) to one part 1,2-dioleoyl-sn-glycero-3-[(*N*-(5-amino-1-carboxypentyl)iminodiacetic acid)succinyl] [nickel salt; DGS-NTA(Ni): product 790404C]} in a glass scintillation vial. The chloroform solvent was removed under a slow and steady flow of nitrogen gas for 2 hours to obtain a dry, thin film in the vial. The film was rehydrated in the kinase buffer [25 mM Hepes-KOH (pH 7.4), 50 mM KCl, 25 mM NaCl, and 5 mM MgCl_2_] to yield liposome sample (25 mg/ml). The buffer was vortexed with the film for 10 min until the lipid was fully dissolved. The aqueous lipid mixture was then subjected to 10 freeze/thaw cycles in liquid nitrogen and a 37°C water bath. The frozen-thawed mixture was then extruded for 11 passes through 100-nm pores of a polycarbonate filter using the Avanti Mini-Extruder. The resulting SUVs were collected in a glass vial and stored at 4°C for up to 1 week. DivJ-His_6_ (5 μM) purified via Ni-NTA affinity purification was incubated in kinase buffer devoid of any lipids for 5 min at room temperature. Following this, serial dilutions of SUV stock solutions were added such that the volume of liposome solution added in each case was the same. As a result of increasing liposome concentrations, the number of available DivJ binding sites per liposome increased. However, since the number of DivJ molecules in the sample is fixed, the increase in the number of available binding sites gives rise to an increased density of DivJ. As a control, SUVs prepared without DGS-NTA lipids were also incubated with DivJ at multiple densities. The incubations were carried out in PCR tubes for 45 min at room temperature. [γ − ^32^P] ATP (1 μCi) was added to each reaction tube, and the tubes were incubated for 2 min at room temperature. Each reaction was performed in triplicate. Each reaction (4 μl) was spotted on the nitrocellulose membrane and dried under a lamp for 40 min. Following the drying, the membrane was washed five times with 50 ml of 12.5 mM sodium pyrophosphate buffer (pH 10). The membrane was dried for 1 hour under an incandescent lamp, wrapped in a plastic sheet, and transferred to a phosphor imaging screen box. The screen was exposed to the membrane for 3 hours followed by phosphor imaging on a Typhoon imager. Details on the estimation of DivJ density on liposomes are included in the Supplementary Text.

#### In vitro phospho-transfer to DivK

To test DivJ’s autokinase activity and its phospho-transfer to DivK, we performed assays in a kinase buffer [50 mM Hepes-KOH (pH 7.4), 25 mM NaCl, 25 mM KCl, and 5 mM MgCl_2_] with equimolar protein concentrations as needed. A total of 5 μM DivJ, 5 μM DivJ, and 5 μM DivK or 5 μM DivJ and 5 μM DivK (D53N) were incubated in the kinase buffer for 30 min at room temperature. The kinase reaction was carried out by the addition of 1 μCi [γ − ^32^P] ATP for 5 min at room temperature. The reactions were quenched by adding 4% SDS followed by separation of proteins by SDS–polyacrylamide gel electrophoresis (SDS-PAGE) in duplicate. One part of the gel was covered in a plastic wrap and transferred to a phosphor imaging screen box. The screen was exposed to the gel for 3 hours followed by phosphor imaging on a Typhoon imager. The other part of the gel was stained using silver chloride and imaged on a protein gel imager.

### Caulobacter protocols

Unless and otherwise stated, *Caulobacter* cells were grown from frozen stocks in PYE overnight with appropriate antibiotics. The following day, cells were diluted in M2G to OD_600_ of 0.01 and grown to OD_600_ of 0.3 to 0.4. Imaging was always performed on agarose pads [composed of 1.5% (w/w) of low melting point agarose (Invitrogen) in M2G].

#### Imaging

For imaging experiments requiring dye labeling, cells growing in M2G in exponential phase (OD_600_ of 0.3) were washed with M2G by centrifugation and then labeled with 5 nM (final concentration) JF549-HaloTag (*12*) by adding a 500 nM stock (in DMSO) solution to the cells. For SpmX-dL5 imaging, cells were labeled with 10 nM (final concentration) of the dye malachite green (MG)–ester (1 μM stock in ethanol and 5% acetic acid) (*41*). The cells were incubated with the dye, shaken for 20 min at room temperature in the dark, and then washed by centrifugation [3 min, 8000 rpm, and 28°C (Eppendorf 5430R)] and resuspension in M2G three times. After the final wash, cells were resuspended in ~20 to 50 μl of M2G, producing a concentrated cell suspension. To this suspension, ~1 nM fiducial markers was added (Molecular Probes, 540/560 carboxylate-modified FluoSpheres; 100 nm in diameter). Next, 1 to 2 μl of the cell/fiducial mixture was deposited onto an agarose pad, mounted onto an argon plasma-etched glass slide (22 mm by 22 mm; no. 1.5, Thermo Fisher Scientific), covered with a smaller (18 mm by 18 mm; no. 1, Thermo Fisher Scientific) plasma-etched slide, sealed with wax, and imaged immediately. For imaging cells with fluorescent proteins, a similar protocol was followed without the dye labeling steps and washing. Cells growing in exponential phase were washed once with M2G and then resuspended in ~20 to 50 μl of M2G. One to 2 μl of this mixture was deposited onto an agarose pad, mounted onto a plasma-etched glass slide, and imaged immediately. For confocal imaging of SpmX-dL5 samples, all steps were carried as above except that no fiduciary markers were used for imaging.

#### In vivo phosphorylation

##### DivJ phosphorylation measurements

In vivo phosphorylation measurements were carried out as described previously (*45*) with the following modifications. Cells from frozen stocks were inoculated into M5G low phosphate medium and grown overnight at 30°C until the OD_600_ was between 0.35 to 0.4. Cultures were normalized to the lowest OD culture in the batch. One milliliter of cells from each culture pulsed with 1 μCi [γ^32^P]-ATP having a specific activity of 30 Ci/mmol (PerkinElmer) for 5 min at room temperature. Immunoprecipitations were performed using protein A agarose beads (Roche). DivJ was immunoprecipitated using an anti-DivJ antiserum (*10*). The immunoprecipitated sample was run on two SDS-PAGE gels. One of the gels was covered in a plastic wrap, dried, and transferred to a phosphor imaging screen box. The screen was exposed to the gel for 5 days followed by phosphor imaging on a Typhoon imager. Proteins separated on the second gel were transferred to a polyvinylidene difluoride (PVDF) membrane, and DivJ levels were measured using Western blots by probing the protein with anti-HaloTag antibody (Promega). Because of the requirement of growing cells in low phosphate media, this assay could not be used to measure phosphorylated DivJ levels under varying glucose conditions.

##### DivK phosphorylation measurements

To measure phosphorylated DivK levels as a function of available glucose, cells were grown in M2 minimal media supplemented with 1 mM, 0.5 mM, or 0.2 mM glucose at 30°C. One milliliter of cells was harvested at an OD_600_ of 0.35 to 0.4. Harvested cells were normalized to the lowest OD_600_ of the collected cells and pelleted by centrifugation at 20,000*g* for 2 min and resuspended in 75-μl lysis buffer [10 mM tris-HCl (pH 7.0), 4% SDS, 5 U deoxyribonuclease I (Thermo Fisher Scientific), and 0.2 tablets of PhosStop (Roche)]. Cell pellets were lysed for 5 min at room temperature. One of the cell pellet samples was lysed at 98°C for 5 min to degrade phosphorylated DivK and serve as a negative control in the assay. After lysis, the samples were spun at 16,000*g* for 5 min at room temperature. Twelve microliters of the supernatant was carefully pipetted from the tubes and mixed with 12 μl of 2× sample buffer [20 mM tris-HCl (pH 7.0), 6% SDS, 10 mM EDTA, 20% glycerol (v/v), and 10% β-mercaptoethanol (v/v)]. Twenty microliters of this sample was loaded on the PhosTag acrylamide gels (FUJIFILM Wako Chemicals) for separation. Phos-tag SDS-PAGE gels were prepared with 50 μM Phos-tag acrylamide and 100 μM ZnCl_2_ and run at 4°C at 80 V. Before transfer by Western blot, gels were washed three times for 10 min in transfer buffer supplemented with 10 mM EDTA at room temperature to remove Zn^2+^ from the gel and washed once with transfer buffer without EDTA. Proteins were transferred using a semidry platform to PVDF membranes at 25 V for 2 hours at room temperature. DivK protein separated on the basis of its phosphorylation state was transferred to a PVDF membrane and detected using a chemiluminescent substrate (SuperSignal West PICO PLUS, Thermo Pierce) after incubation with mouse anti-Flag (1:10,000) primary antibody (Sigma-Aldrich, F1804) and a polyclonal goat anti-mouse horseradish peroxidase (HRP; 1:10,000)–conjugated secondary antibody (Abcam). Phosphorylated and nonphosphorylated forms of DivK were estimated using Fiji from three biological replicates.

#### In vivo chemical perturbations

To test the effect of different chemical perturbations on fluorescently tagged proteins in vivo, *Caulobacter* cells were grown from frozen stocks in PYE to an OD_600_ of 0.3 to 0.4 (or 0.2 for ∆*popZ* strains). SpmX and DivJ clusters under native expression exhibited low fluorescence signal, which often obscured small differences between cluster intensities observed under chemical perturbations. To observe reproducible differences in protein cluster intensities, protein production was induced for 4 hours by supplementing the growth media with 0.3% xylose. After induction, 1 ml of cells was spun down (12,000*g* for 2 min) and resuspended in M2G (for CCCP treatment) or M2 minimal media (for LA or 1,6-HD treatment). A total of 100 μM CCCP (10 min), 5 μM LA (30 min), or 5% 1,6-HD (30 min) were added to the resuspended cultures followed by shaking at 30°C for the indicated amount of time. After the treatment, 1 to 2 μl of cell samples were deposited onto an agarose pad, mounted onto a glass coverslip, and imaged immediately. Then, 80-μm fields of view were imaged per sample, and this routine was repeated for three biological replicates. For measuring cell health under solute treatment, a cell impermeant fluorescent dye (Live-or-Dye 640-662, Biotium) was used. Lyophilized dye powder was reconstituted in 50 μl of anhydrous DMSO based on the manufacturer’s protocols. One microliter of the dye stock was added to 1 ml of cells treated with various perturbations, and the samples were incubated on a rotatory shaker at 30°C for 30 min. As a control, an equivalent volume of DMSO was added to untreated cells. Cells were washed thrice with 1 ml of M2G media followed by imaging on agarose pads. Cells that sequestered the dye within them were at least fivefold brighter than the healthy cells. Accordingly, a threshold was applied to obtain the number of cells with or without membrane leakage using bespoke code in MATLAB.

#### Glucose-dependent measurements

##### Cell length

For cell length measurements, cell cultures were started from frozen stocks in PYE media. These cultures were collected in exponential phase and washed once with M2 minimal media without glucose. Cells were diluted to an OD_600_ of 0.05 in respective media [M2G (1 mM), M2G (0.5 mM), etc.] in triplicate and grown to exponential phase (OD_600_ of 0.2 to 0.4). These cultures were diluted 10-fold and imaged on M2G agarose pads immediately. OD_600_ for each culture was measured, 1 ml of each culture was centrifuged at 16,000*g* for 2 min, and the medium was aspirated. These pellets were frozen in liquid nitrogen and were stored at −80°C before ATP measurements. For intracellular ATP measurements, the pellets were thawed and resuspended in 300 μl of M2 minimal media, and the assay was performed as per the manufacturer’s protocols (BacTiter-Glo, Promega) (*37*). For each sample, luminescence measurements were made in three wells, and average value was taken from nine wells per strain and condition (for example, WT strain in M2G had pellets from three biological replicates, each pellet was resuspended in 300 μl of lysis buffer and was split into three samples of 100 μl per sample for the luminescence assay). Cell length analysis was performed using MicrobeJ plugin for Fiji (*46*, *47*). We used manual mode detection for long and filamentous cells [representing SpmXΔIDR, ΔSpmX, or DivJ(H338A) phenotypes] that could not be selected by the automated detection using a reasonable set of thresholding parameters.

##### Growth curve

For growth curve measurements, cells in exponential phase were back diluted to OD of 0.02 and grown in 96-well plates at 28°C with constant shaking in between measurements on a Tecan Safire multiwell plate reader. Absorbance at 600 nm was measured every 10 min.

#### Western blots

To assess the levels of SpmX in different strains (fig. S2H), cells were grown in M2 minimal media supplemented with 1 mM glucose at 30°C. Cells were incubated with or without indicated concentrations of xylose for 30 min to enable protein induction. One milliliter of cells was harvested at an OD_600_ of 0.35 to 0.4. Harvested cells were normalized to the lowest OD_600_ of the collected cells, pelleted by centrifugation at 20,000*g* for 2 min, and resuspended in 30 μl of 2× sample buffer [20 mM tris-HCl (pH 7.0), 6% SDS, 10 mM EDTA, 20% glycerol (v/v), and 10% β-mercaptoethanol (v/v)] and incubated at 98°C for 10 min. Twenty microliters of this sample was loaded on Mini-PROTEAN Precast Mini PAGE Gels (Bio-Rad) for separation. Proteins separated via SDS-PAGE were transferred using a semidry platform to PVDF membranes at 80 V for 1 hour at room temperature. SpmX levels on the PVDF membrane were detected using a chemiluminescent substrate (SuperSignal West PICO PLUS, Thermo Pierce) after incubation with an anti-SpmX (1:10,000) primary antibody raised against the SpmX-IDR (*7*) and a polyclonal goat anti-mouse HRP (1:10,000)–conjugated secondary antibody (Abcam). SpmX levels were estimated using Fiji from three independent biological replicates.

### Microscopy

#### Diffraction-limited wide field imaging (phase contrast and fluorescence)

##### Data acquisition

Cells immobilized on agarose pads or in vitro samples in multiwell glass bottom plates were imaged on a light-emitting diode–based (Lumencor, SpectraX) multicolor epifluorescence microscope consisting of a Leica Dmi8 stand equipped with an immersion oil phase contrast objective [100×, HC PL APO, 1.4 numerical aperture (NA)] and an EMCCD camera (Hamamatsu, C9100 02 CL). A list of all the filters used is provided in table S2.

##### Data analysis

 Analyses for condensate intensity, either at a single time point or as a time series, were performed using bespoke software written in MATLAB. Cell length analyses were performed using MicrobeJ (*46*). Analyses of fluorescent clusters in live cells were performed using bespoke software in MATLAB. A binary mask was generated using the phase contrast images of the cells. In each cell within the mask, a threshold was applied to obtain all clusters within a cell. The mean fluorescence intensity from a cellular region not containing the clusters was subtracted from the mean fluorescence intensity of the clusters. This number was divided by the mean fluorescence intensity of the cell for normalization. The automated threshold did not work robustly for RNase E–eYFP and DivJ-eYFP cells under conditions where the cluster intensities were very low. In these cases, a manual thresholding step was added on top of the phase contrast–based threshold. For two-color analyses (fig. S4A), a binary mask was created using the phase contrast images followed by thresholding the SpmX channel to detect and quantify fluorescent clusters. The thresholded image for SpmX channel was then converted to a binary mask and applied to the DivJ channel. Fluorescence value calculations and normalization were performed as above.

#### Confocal imaging and FRAP

##### Data acquisition

Confocal microscopy was used for imaging in vitro condensates and *E. coli* cells. Samples were imaged at room temperature on a spinning disk confocal microscope (Leica DMI6000B custom-adapted with a Yokogawa CSU-X1 spinning disk head; a Photometrics Evolve 512 EMCCD camera; and Intelligent Imaging Innovations SlideBook software, Vector FRAP, LaserStack), with a 100× oil objective (HCX Pl APO, 1.4 NA; Leica). eYFP fluorescence was imaged by excitation at 514 nm and emission with a YFP 540/15 filter (Semrock) and 445/514/561-nm Yokogawa dichroic beam splitter (Semrock) under the following conditions: 100-ms exposure every 1 s for 100 time points, with <2-mW laser power (measured at the fiber), with Adaptive Focus Control active during the entire acquisition. Vector was used to direct the 514-nm laser at full power for photobleaching (∼18 mW, measured at the vector fiber), which took place between the third and fourth image captures on a region of interest positioned at the center of each selected condensate. Line mode of photobleaching was also used on *Caulobacter* and small condensates.

##### Analysis

FRAP images were exported as tiff files into a MATLAB workspace. Image background was measured from regions that did not have any condensates, averaged, and subtracted from the image. FRAP analyses protocol was developed based on reports elsewhere (*48*). Three regions of interest were selected using the *roipoly* function in MATLAB. Region 1 was the bleached region within a condensate, region 2 was a control region on another condensate that was not bleached, and region 3 was the entire bleached condensate. Intensity as a function of time was measured from all the three regions. Intensity from region 2 was subtracted from region 1, and the resulting intensity was divided by the intensity from region 3 to normalize for laser/sample fluctuations. Last, the resulting signal was normalized such that the prebleach signal was set to 1, and the resulting FRAP curves were reported as fractional recovery curves.

#### Single-molecule imaging

##### Single-molecule imaging and data acquisition

Cells immobilized on agarose pads were imaged on the two-color home-built epifluorescence microscope described previously (*11*). 2D white light transmission images of the cells were recorded before imaging. Fluorescence emission from the sample was collected through a high NA oil immersion objective (Olympus UPlanSApo 60×/1.40 NA) and then filtered by a multipass dichroic mirror (Chroma, zt440/514/561/642rpc). For detecting eYFP, a 514-nm long-pass filter (Semrock LP02-514RE) and a bandpass filter (Semrock FF01-523/610) were used. For detecting JF549, a 560-nm dichroic beam splitter (Semrock FF560-FDi01), a 561-nm notch filter (Semrock NF03-561E), and a bandpass filter (Semrock FF01-523/610) were used. eYFP was pumped with 514-nm excitation (MPB Communications; fiber laser of 514 nm, 1 W, ~0.01 to 0.1 kW/cm^2^). JF549 was excited with a 561-nm laser (Coherent Sapphire 561 100 CW, ~0.75 kW/cm^2^) and reactivated with a 405-nm laser (Coherent Obis; ~0.1 to 10 W/cm^2^). Camera integration time was 20 ms.

##### Single-molecule data fitting

3D single-molecule data were fit using Easy-DHPSF v2.0, freely available at https://sourceforge.net/projects/easy-dhpsf/ (*49*). Calibration scans were generated by axially scanning a fluorescent bead (540/560, 100-nm FluoSpheres; Life Technologies) using a piezoelectric stage scanner (Physik Instrumente P-545). This axial calibration scan was used to relate angular lobe orientation to axial position and to generate templates needed to locate candidate single molecules in the data. Background photons [~7 ± 1 photons per pixel (means ± SD)] were estimated using a temporal median filter. On average, we detected 958 ± 354 photons (means ± SD) per 20-ms frame for the DivJ-Halo-JF549 molecules (561-nm laser intensity of ~0.75 kW/cm^2^). Localization precision was estimated with an empirical formula derived from repeatedly localizing single beads under variable background conditions (*11*). Localizations used for SPT analysis had typical localization precisions of 30.4 ± 7.5 nm, 31.5 ± 8.0 nm, and 46.9 ± 11.7 nm in *x*, *y*, and *z*, respectively (means ± SD). Systematic errors from sample drift resulting from mechanical and thermal fluctuations were accounted for by adding ~1 nM concentration of 540/560, 100-nm FluoSpheres (Life Technologies) to the sample and using these as fiducial beads, whose motion was removed from the single-molecule localization data. Diffusion analyses are included in the Supplementary Text.

#### Airy scan confocal microscopy

##### Data acquisition

Two-color superresolution imaging of condensates or live *Caulobacter* cells (Fig. 4) was performed on an inverted laser scanning confocal microscope (Zeiss LSM880) equipped with an AiryScan module (*50*). AiryScan detector alignment was performed as per the Zeiss manual in both channels. Condensate imaging (Fig. 2F) was performed using a PLAN APO 63×, 1.4 NA oil immersion objective. Atto488 was excited using a 488-nm laser (at 2%), and the resulting fluorescence was collected within a detection window spanning 509 to 541 nm. Cy3 was excited using a 561-nm laser (at 7%), and the resulting fluorescence was collected within a detection window spanning 570 to 610 nm. Live cell imaging (Fig. 4B) was performed using an EC PLAN NEO 40×, 1.3 NA oil immersion objective. mCherry was excited using a 561-nm laser (at 1.9%), and the resulting fluorescence was collected within a detection window spanning 570 to 620 nm. dL5 was excited using a 633-nm laser (at 0.5%), and the fluorescence was collected using a 645-nm long-pass filter.

##### Analyses

Analyses of condensate demixing in vitro were performed using bespoke software written in MATLAB. For each image, a binary mask was generated by applying an intensity threshold on the PopZ channel. The mask was applied on the SpmX channel followed by the application of a spot detection algorithm to count the number of SpmX clusters. A similar analysis was performed for live-cell images whereby PopZ assemblies above a set intensity threshold and larger than 1 μm were used to create a binary mask. This mask was applied on the SpmX channel followed by the spot detection analyses as in the previous case, to count the number of SpmX clusters within PopZ microdomain.

#### Correlative cryogenic electron microscopy

Cryogenic single-molecule imaging of SpmX-PAmKate fusions expressed in live *Caulobacter* cells was performed according to protocols reported previously (*51*). SpmX localizations from four cells were pooled and overlaid on a representative image of *Caulobacter* obtained from cryo–electron tomography. Registration between single-molecule fluorescence and cryo–electron microscopy images was performed as reported previously (*23*, *52*–*56*).
