# Supplementary material for: ATP-responsive biomolecular condensates tune bacterial kinase signaling
Source: Sci Adv. 2022 Feb 16;8(7):eabm6570. doi: 10.1126/sciadv.abm6570 (PMC8849385; doi:10.1126/sciadv.abm6570)
Supplement: Supplementary file 1 — Supplementary text Figs. S1 to S4 Tables S1 and S2 [file sciadv.abm6570_sm.pdf]

Science Advances
AAAS

Supplementary Materials for

ATP-responsive biomolecular condensates tune bacterial kinase signaling

Saumya Saurabh*, Trisha N. Chong, Camille Bayas, Peter D. Dahlberg, Heather N. Cartwright, W. E. Moerner, Lucy Shapiro*

*Corresponding author. Email: saumyas2@stanford.edu (S.S.); shapiro@stanford.edu (L.S.)

Published 16 February 2022, *Sci. Adv.* **8**, eabm6570 (2022)
DOI: 10.1126/sciadv.abm6570

The PDF file includes:

Supplementary text
Figs. S1 to S4
Tables S1 and S2
Legends for movies S1 to S5

Other Supplementary Material for this manuscript includes the following:

Movies S1 to S5

Supplementary text

Assessment of DivJ diffusion using mean squared displacement

Single-molecule trajectories of DivJ were constructed by connecting localizations from consecutive frames. Only trajectories of at least 9 steps (10 frames or 200 ms) were used for MSD analyses. The maximum allowed displacements of DivJ-Halo-JF549 molecules over 20 ms were set to 750 nm. For DivJ-Halo-JF549, we detected a median trajectory length of 17 frames (0.34 s). Diffusion coefficients were calculated from ensemble-averaged 3D MSDs (Equation 1) (*52-54*) of individual DivJ molecules using only the first two time lags to avoid long-time effects of confinement and non-Brownian motion. The reported diffusion coefficient errors are diffusion coefficient standard deviations estimated by bootstrapping (50% of the trajectories sampled with replacement 500 times).

$$\mathrm{MSD}(\tau) = 2dD\left(\tau - \frac{\Delta t}{3}\right) + 2\sum_{j=1}^{d}\sigma_j^{\,2} \qquad \text{Equation 1}$$

In Equation 1 above, τ is time lag, Δt is camera exposure time, d is the number of dimensions (3 in this case), D is the diffusion coefficient, and σ_j is the localization error in dimension j.

To distinguish between diffusion behaviors for polar trajectories, a cumulative distribution function (CDF) of all displacements in 20ms intervals was calculated (*13*). Since the jumps within this time lag were less than three times the localization precision of the measurements, this procedure could not be used for extracting the diffusion coefficients. To further validate CDF assessments of DivJ trajectories, mean-squared-displacement (MSD) analyses were performed. MSD analyses also indicated that DivJ diffusion was on average ten-times faster in the polar microdomain in the absence of SpmX-IDR (Fig. S1C). As a negative control, we engineered a HaloTag fused to the trans-membrane helical region from an *E. coli* sensor protein ArcB, such that the resulting fusion does not interact with SpmX or any other proteins in *Caulobacter*. The membrane-localized HaloTag displayed the same diffusivity in the pole in the presence or absence of SpmX (Fig. S1D), indicating that the differences in diffusion observed for DivJ are specific to its interaction with SpmX. Cumulatively, single molecule diffusion analyses indicate that DivJ is localized to the stalk-bearing pole through an interaction that is facilitated by the SpmX-IDR, resulting in a higher polar dwell time and sequestration of DivJ.

eYFP fusion of SpmX-IDR

SpmX, on account of containing structured and disordered domains, exhibits oligomerization and multivalency that is dependent on both its domains (*8*). Accordingly, we observed that SpmX-IDR

(AA156-355) was unstable in E. coli and as a result could not be purified to concentrations above 5 µM. Likely, this was a result of the SpmX-IDR getting proteolyzed in E. coli due to its exposed disordered termini. Several strategies were used to "cap" the N-terminus of the IDR, including 6xHis-Tag, 3xFlag-Tag and an eYFP-fusion. While the His-tagged and Flag-tagged proteins exhibited meagre improvements in yield, the eYFP fused IDR could be purified at high concentrations (~25 µM). Despite the improved yield eYFP-IDR condensates were unstable under imaging conditions, and they collapsed and degraded upon touching the glass surface. As a result, they were imaged while diffusing above the glass surface. This imposed a challenge in two-color imaging of eYFP-IDR and DivJ (sparsely labeled with Cy3), which was overcome by 3D imaging of eYFP-IDR condensates in the presence of DivJ (Movies S1-2). Enrichment of DivJ signal was observed in all eYFP-IDR condensates, indicative of a direct interaction between SpmX-IDR and DivJ *in vitro*. This observation could also explain the SpmX-IDR dependent dwell time exhibited by DivJ in the polar microdomain, measured via single protein tracking.

PopZ gelation states

Upon imaging PopZ that was reconstituted from frozen protein stocks, we observed non-spherical condensates reminiscent of a "beads on a string" morphology. Such morphology has been observed for thermally trapped poly-Arginine-RNA condensates (*20*). To understand whether PopZ condensates with beads on a string morphology represented thermally trapped states, we heated the reconstituted samples prior to imaging. The beads on a string morphology of PopZ condensates transitioned to a spherical morphology after heating the samples at 95°C for 8 mins. Spherical PopZ condensates formed after heat treatment sequestered SpmX *in vitro*, suggesting that SpmX binding was not impacted via thermal treatment. PopZ within spherical condensates exhibited slower internal rearrangements compared to aspherical condensates (Fig. S2I). Spherical condensates fused, while aspherical maintained their beads on a string morphology over a two hour observation window (Fig. S2J). Together, the distinct morphologies of PopZ condensates represent different states on the PopZ phase diagram *in vitro*. The relevance of these morphological phase transitions of PopZ condensates *in vivo* remains to be understood.

DivJ kinase assay on liposomes

The estimation of number of DivJ molecules per liposome is based on similar experiments with the protein kinase CckA (*44*). The goal of this estimation was to assess whether the number of DivJ molecules per liposome is limited by geometrical considerations (area) or concentration of

protein and Nickel-NTA sites. To estimate the number of available binding sites for DivJ molecules on a liposome, we calculated the total surface area of the liposome. DOPG liposomes extruded through 100 nm pores have vesicle diameters of 97-106 nm (*55*), that we approximated as 100 nm for simplification of calculation. Based on these calculations, we estimated that at an equal mass of lipid to DivJ protein, the density of DivJ was ~1100 molecules per liposome. Further calculations of DivJ density were made based on appropriate dilutions. The data from liposome spot assays in triplicate was averaged and fit using the Hill-Langmuir equation:

$$P = \frac{[DivJ]^n}{K_d + [DivJ]^n} \qquad \text{Equation 2}$$

In equation 2, P denotes the fraction of DivJ phosphorylated, denoted by the end point kinase activity, $[DivJ]$ denotes the estimated DivJ density, K_d is the apparent dissociation constant, and n is the Hill coefficient.

Supplementary Figures

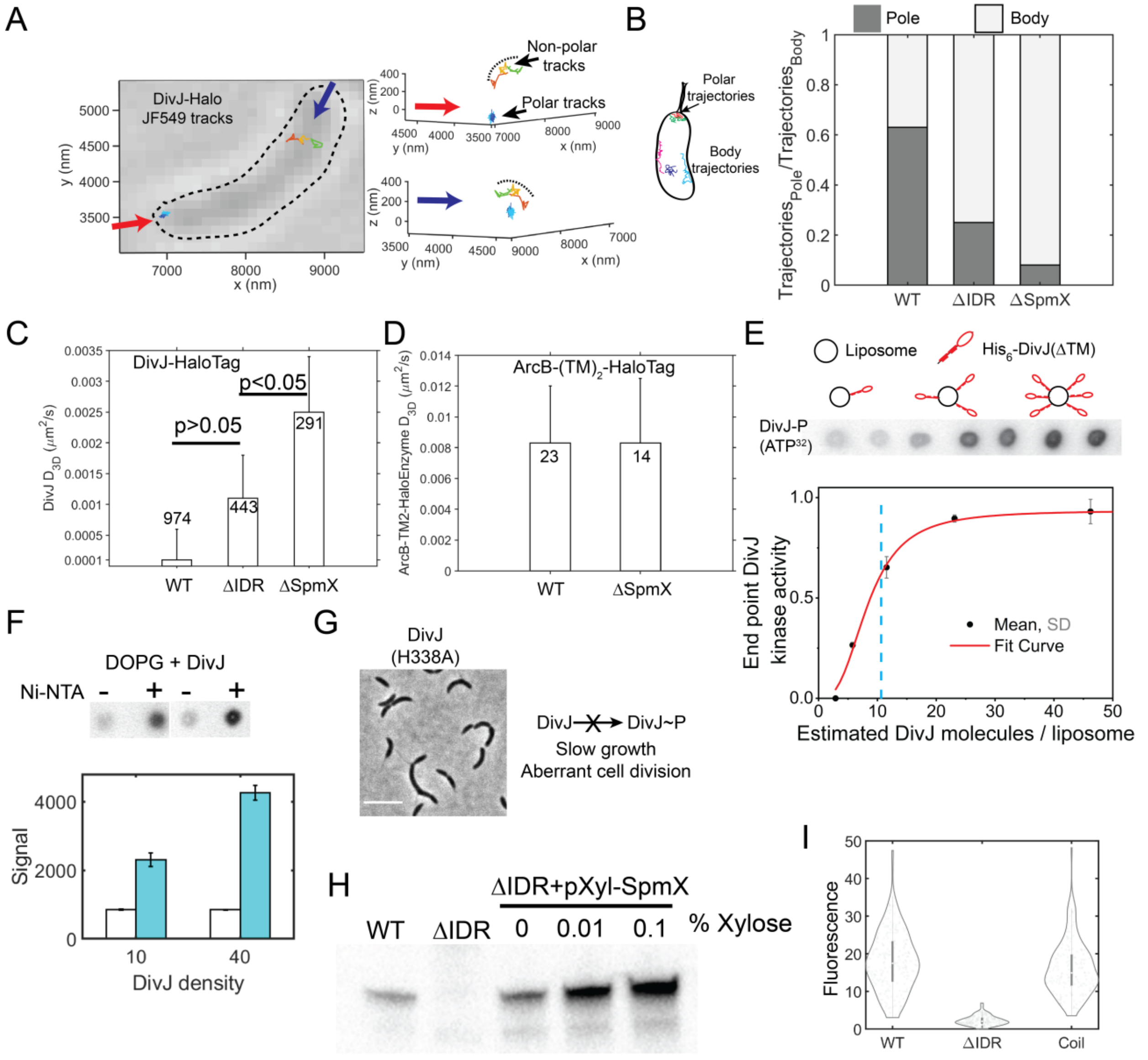

Fig. S1. Effect of SpmX-IDR on DivJ localization, diffusion, and activity. Throughout this panel, ΔIDR denotes SpmXΔIDR. **(A)** (left) Representative bright-field image of a live *Caulobacter* cell, overlaid with 2D projections of 3D DivJ-HaloTag trajectories measured using single-particle tracking. Each trajectory is plotted using a different color. Arrows denote perspectives for the 3D representation shown on the right. (right) Representation of the trajectories from the left panel in 3D space with appropriate perspective and all three Cartesian axes. Green and red trajectories in the body are membrane-associated as can be seen from their curvature, highlighted using a black dashed line. 3D tracking reveals polar as well as non-polar membrane

associated trajectories. **(B)** Fraction of single molecule DivJ trajectories observed in the cell pole vs the body for respective strains. Total tracks analyzed are 1558 (WT), 1764 (ΔIDR) and 3572 (ΔSpmX). SpmX-IDR promotes DivJ sequestration at the pole. **(C)** Three-dimensional diffusion coefficients obtained from Mean Squared Displacement (MSD) analyses for polar DivJ trajectories in various SpmX backgrounds. The number of trajectories analyzed for each sample are noted with the bars. P values from a pairwise two-tailed t-test are denoted in the plot. **(D)** 3D diffusion coefficients obtained from MSD analyses of the control trans-membrane HaloTag trajectories in WT and SpmX deletion backgrounds. The HaloTag was attached to the membrane using two trans-membrane helices from the *E. coli* protein ArcB. Differences were not detected in the polar diffusion coefficients of the HaloTag in WT or SpmX deletion backgrounds. Errors in D in both panels (C) and (D) are standard deviations estimated from bootstrapping (50% sampling with replacement performed 500 times). **(E)** DivJ auto-kinase activity assayed on liposomes. Liposomes contained 90% (mol %) di-oleoyl-phosphatidyl glycerol (DOPG) and 10% (mol %) Nickel-chelated lipids (DGS-NTA) to bind His_6-DivJ(ΔTM). A fixed concentration of 5 μM His_6-DivJ(ΔTM) was pre-incubated with increasing amounts of liposomes to obtain increasing surface density of His_6-DivJ(ΔTM) molecules per liposome. Kinase reaction was performed in a buffer (25 mM HEPES-KOH, pH 7.4, 50 mM KCl, 25 mM NaCl, 5 mM $MgCl_2$) by adding 0.1 μCi ATP(γ–^{32}P) for 5 min, quenching and blotting on a nitrocellulose membrane followed by phosphor imaging. The measured activity at each His_6-DivJ(ΔTM) surface density state was normalized to the highest density condition. Representative phosphor imaging data from spot assays and a schematic depicting increasing DivJ density (red) on liposomes (black circles) are shown above the graph. Intensity from phosphor imaging spots was quantified and the average data from triplicate spot assays were fit to a Hill-Langmuir curve as described in the supplementary text. Dashed blue line represents the average physiological polar DivJ concentration of 11 molecules measured from a single molecule counting assay for cells grown in M2G media. Error bars represent the standard deviation from 3 measurements. DivJ exhibited a density dependent kinase activity *in vitro*. **(F)** Liposome phosphorylation assay with (cyan bars) or without (white bars) Ni-NTA lipids at two different estimated densities of 10 and 40 DivJ molecules/ liposome. Error bars are standard deviations from 3 measurements. DivJ signal from liposomes without Ni-NTA lipids is insensitive to the protein density. **(G)** Representative phase contrast image of DivJ (H338A) cells in which DivJ phosphorylation is absent, leading to aberrant cell division (scale bar 5μm).

(H) Western blot showing the levels of WT SpmX in WT, SpmXΔIDR, and SpmXΔIDR cells expressing WT SpmX on a chromosomal Xylose promoter (rescue cell line). For the rescue cell line, WT SpmX was produced by adding the indicated amount of Xylose. Western blot was performed using an antibody raised against the IDR of SpmX. Even without Xylose addition, the mild expression of SpmX was comparable to SpmX levels in WT cells. **(I)** Distribution of the ratio of mean polar and non-polar DivJ signal in WT cells (N=235), ΔIDR cells (N=258) and cells with SpmXΔIDR fused to a CoilZ peptide (N = 204) that can bind to a cognate CoilY peptide fused to DivJ-eYFP. Coil tag recruitment of DivJ gives a polar signal distribution comparable to WT cells independent of the SpmX-IDR.

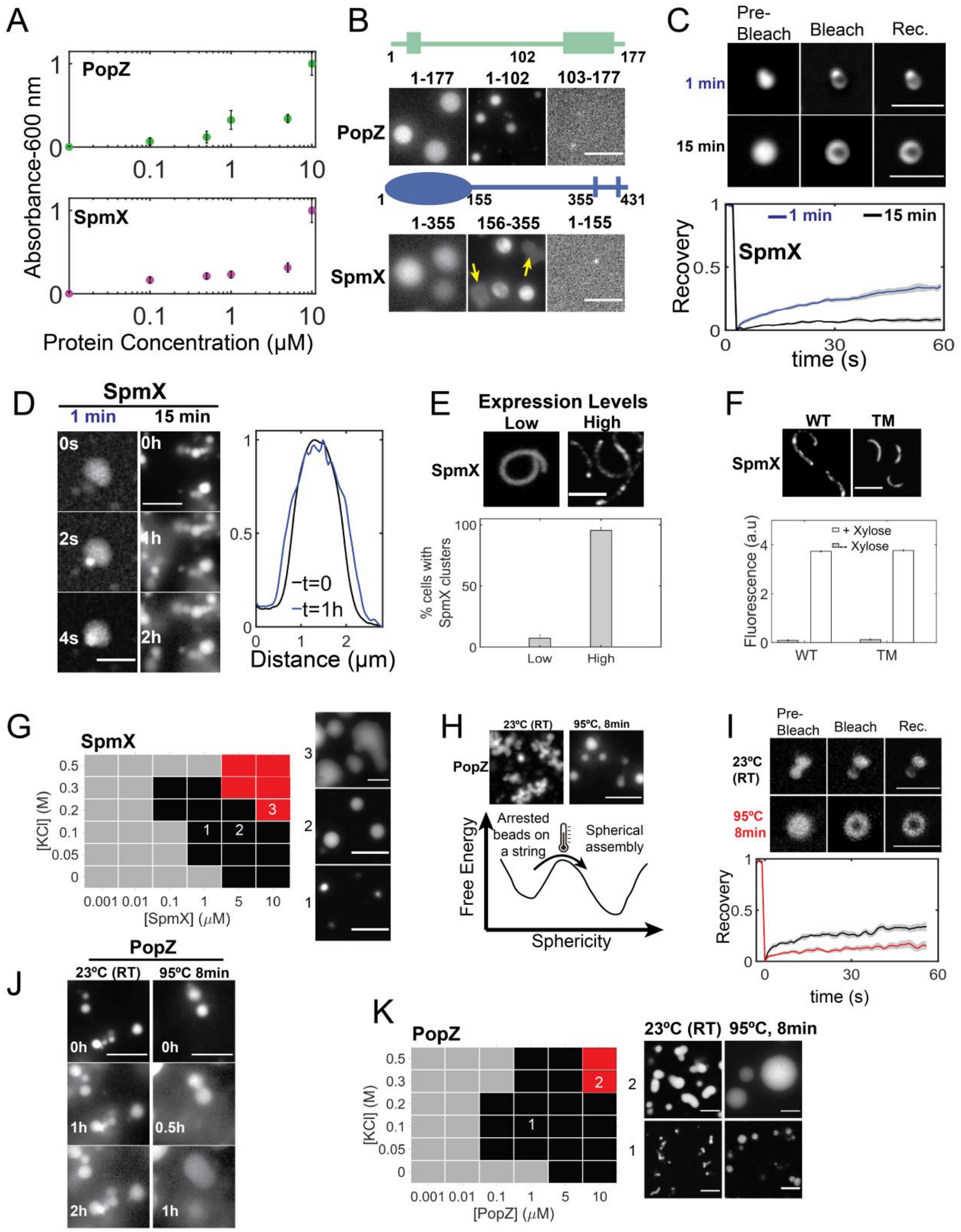

Fig. S2. Context dependent properties of SpmX and PopZ condensates. (All scale bars in this figure are 5 µm.) **(A)** Solution turbidity measured as a function of increasing PopZ (top) or

SpmX(ΔTM) (bottom) concentration in a physiological buffer (50 mM HEPES-KOH, pH 7.4, 0.1 M KCl) at room temperature. Background subtracted-absorbance values were normalized to the maximum absorbance observed in each case. Error bars represent standard deviation from 3 measurements. PopZ and SpmX exhibit a concentration dependent increase in turbidity. **(B)** (top) Domain diagram and representative fluorescent micrographs of PopZ domains: PopZ (full length), PopZ (AA 1-102) and PopZ (AA 103-177) (all proteins 1% Atto488 labeled). (bottom) Domain diagram and representative fluorescent micrographs of SpmX domains: SpmX (ΔTM, 5% Cy3 labeled), eYFP-tagged SpmX-IDR, and SpmXΔIDR (ΔTM, 5% Cy3 labeled). Yellow arrows in eYFP-tagged SpmX-IDR sample indicate condensates that degraded upon contacting the coverslip. All proteins are at 5 µM concentration. IDR containing regions of both SpmX and PopZ are necessary and sufficient for phase separation *in vitro*. **(C)** Internal rearrangement of SpmX condensates was assayed by FRAP at two different time points after fusing on glass. (top) A 250 nm spot was bleached in a SpmX condensate (5 µM protein, ΔTM, 5% Cy3 labeled), and the change in the fluorescence was analyzed as a function of time for condensates within 1 min (blue) or 15 mins (black) of relaxing on glass. (bottom) Analyses of the fluorescence recovery of 1-min-old (blue; N = 9), 15-min-old (black; N = 11) condensates are shown. Gray shadow depicts the standard error of the mean. SpmX condensates exhibit concentration dependent dynamics *in vitro*. **(D)** (Left) Condensates of SpmX fused to glass within 1 min (5 µM protein, ΔTM, 5% Cy3 labeled) displayed spontaneous fusion on the seconds time scale, and (Right) condensates fused to glass longer than 15 mins ripen on the hours' time scale without fusion. Measurement of the diameter of a ripening SpmX condensate over 1 hour showed an increase in its diameter by ~350 nm. SpmX ripening is associated with slow condensate internal dynamics likely due to protein self-association. **(E)** Fluorescence micrographs of *Caulobacter* cells harboring a *popZ* deletion and over-expressing eYFP-labeled SpmX on a chromosomal Xylose promoter, induced using 0.03% Xylose (left) or 0.3% Xylose (right) for 1 hour. Bar graph below shows the % of cells exhibiting two or more SpmX clusters per cell (N ~ 400 cells for each case). SpmX clusters observed under 0.03% Xylose induction were approximately 3-4 times less intense compared to clusters observed under 0.3% Xylose induction. SpmX cluster formation is concentration dependent *in vivo*. **(F)** Representative fluorescence micrographs of cells expressing SpmX-eYFP in a *popZ* deletion (left) or SpmX(TM)-eYFP (Δ1-355) in a *spmX* deletion, on a chromosomal Xylose promoter, induced using 0.3% Xylose for 1 hour. While clusters were observed for WT SpmX (>95% cells), SpmX-

TM (Δ1-355) always remained diffuse. Shown below are end point YFP fluorescence measured using a plate reader in cells expressing SpmX-eYFP or SpmX-TM-eYFP (Δ1-355) grown in the presence or absence of 0.3% Xylose for 1 hour. At comparable concentrations, SpmX-TM domain without the cytoplasmic domain was unable to form clusters *in vivo*. **(G)** Phase diagram of SpmX as a function of protein and KCl concentration. Black squares denote conditions under which >20 fluorescent spots (> 2 μm) were observed to be above the intensity threshold (<background> + $3\sigma_{background}$) from 80 μm square regions across six fields of view spanning two biological replicates. Red squares denote metastable condensates suggestive of the spinodal decomposition on the phase diagram. Representative fluorescent micrographs from conditions 1-3 are shown next to the plot. **(H)** Representative maximum intensity projections of 3D confocal micrographs of PopZ (5 μM, 1% Atto 488 labeled) reconstituted in a physiological buffer (50 mM HEPES-KOH, pH 7.4, 0.1 M KCl). PopZ condensates exhibited "beads on a string" morphology at 23 ˚C (left) but relaxed into spherical condensates upon heat treatment (right). This suggests the presence of a thermal barrier between conformational states of different sphericity, depicted in the schematic below. **(I)** Internal rearrangement of PopZ condensates (5 μM, 1% Atto488 labeled) *in vitro* was assayed by FRAP. (top) A 250 nm spot was bleached, and the change in the fluorescence was analyzed as a function of time for untreated condensates (black) and heat treated condensates (red). (bottom) Analysis of fluorescence recovery of untreated condensates (black; N = 10), heat treated condensates (red; N = 12) are shown. Gray shadow depicts the standard error of the mean. Heat treated condensates (spherical) exhibit slower internal dynamics compared to arrested (aspherical) condensates. **(J)** Representative fluorescence micrographs showing ripening behavior of arrested PopZ condensates (5 μM protein, 1% Atto488 labeled) without fusion on the hours' time scale *in vitro*. Heat treated PopZ condensates fused over sub-1 hour time scales showing the differences between the slow dynamic states of condensates *in vitro*. **(K)** Phase diagram of PopZ condensates (1% Atto488 labeled) as a function of protein and KCl concentration. Black squares denote conditions under which >20 fluorescent spots (> 2 μm) were observed to be above the intensity threshold (<background> + $3\sigma_{background}$) from 80 μm square regions across five fields of view spanning two biological replicates. Red squares denote metastable condensates suggestive of a spinodal decomposition on the phase diagram. Representative fluorescent micrographs from conditions 1-2 for the untreated and heat treated samples are shown next to the plot.

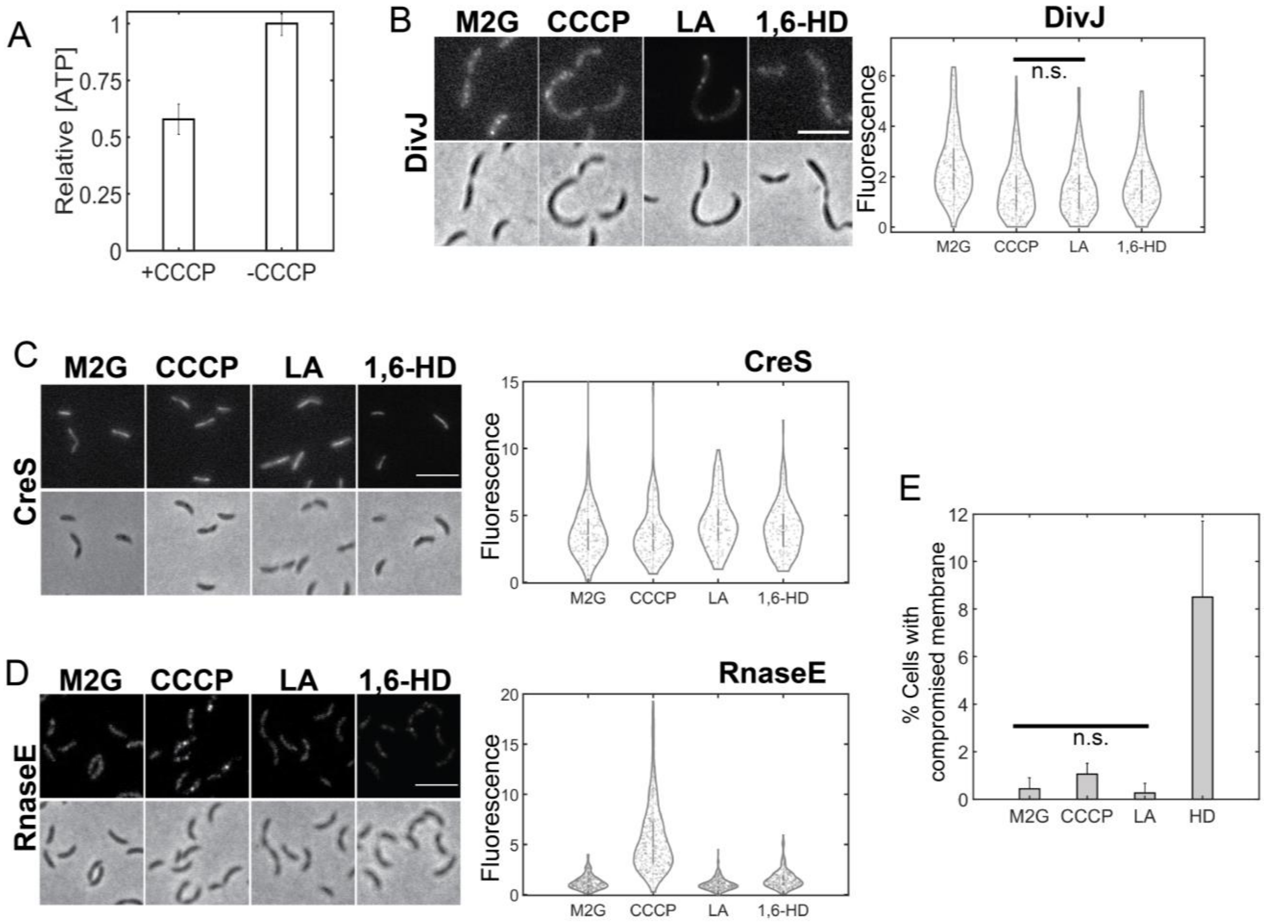

Fig. S3. Effect of solutes on various protein assemblies and membrane integrity *in vivo*. (All scale bars in this figure are 5 µm.) **(A)** Relative intracellular ATP concentrations in *Caulobacter* cells treated with 100 µM CCCP dissolved in DMSO, or an equivalent volume of DMSO for 10 minutes, measured using a commercial Luciferase based assay. Background from the cell growth media was subtracted and luminescence units were normalized to the ATP levels in the DMSO (-CCCP) case. Error bars represent the standard deviation from 3 biological replicates, with each sample assayed in triplicate. CCCP addition leads to rapid ATP depletion in live *Caulobacter* cells. **(B)-(D)** Effect of ATP depletion by CCCP addition (100 µM, 10 min), 5 µM LA, or 5% (v/v) 1,6-HD treatment (30 mins, each) on various protein assemblies *in vivo*. Differences between all distributions are statistically significant ($p < 0.0005$) unless connected by a line denoting n.s. ($p > 0.0005$, based on a two-sample t-test). **(B)** Representative fluorescence (top) and phase contrast (bottom) micrographs of *Caulobacter* cells with *spmX* deletion and over-expressing DivJ-eYFP on a high copy Xylose inducible plasmid exhibited fluorescent clusters. However, the fluorescence

within DivJ-eYFP clusters was depleted under all treatments (CCCP, LA, 1,6-HD). ~600 cells were analyzed for each condition. **(C)** Representative fluorescence and phase contrast micrographs of *Caulobacter* cells over-expressing Cres-eYFP exhibited fluorescent fibers. The relative fluorescence intensity in these fibers depleted under CCCP treatment, while a mild enhancement in fluorescence was observed under 1,6-HD or LA treatment. ~500 cells were analyzed for each condition. **(D)** *Caulobacter* cells expressing endogenously tagged RnaseE-eYFP exhibited clusters with enhanced fluorescence under CCCP treatment (10 mins) and depleted fluorescence under LA treatment but not under 1,6-HD treatment (30 mins, each). ~800 cells were analyzed for each condition. ATP and LA, but not 1,6-HD, are able to dissolve condensates such as BR bodies while having distinct effects on structured protein assemblies such as CreS, or protein aggregates such as DivJ in the absence of SpmX *in vivo*. **(E)** Bar plot showing percentage of *Caulobacter* cells expressing eYFP-PopZ with membrane leakage observed under treatment with CCCP (N = 762 cells), LA (N=682 cells) and 1,6-HD (N = 474 cells), compared with the untreated cells (M2G, DMSO, N = 719 cells). Cells were treated with the respective solutes followed by labeling with Live-or-Dye™ (640/662) dye based on manufacturer's protocols. Dye labeled cells were washed thrice using M2G and imaged on an epifluorescence microscope. Cells with compromised membranes exhibited at least five-fold higher fluorescence compared to healthy cells. This threshold criterion was applied to all the images to obtain the bar plots. Error bars are standard deviation across 12 fields of view from three biological replicates. n.s. denotes $p > 0.05$ based on a two sample t-test. 1,6-HD treated cells exhibited 4-5-fold higher membrane leakage than CCCP or LA treated cells.

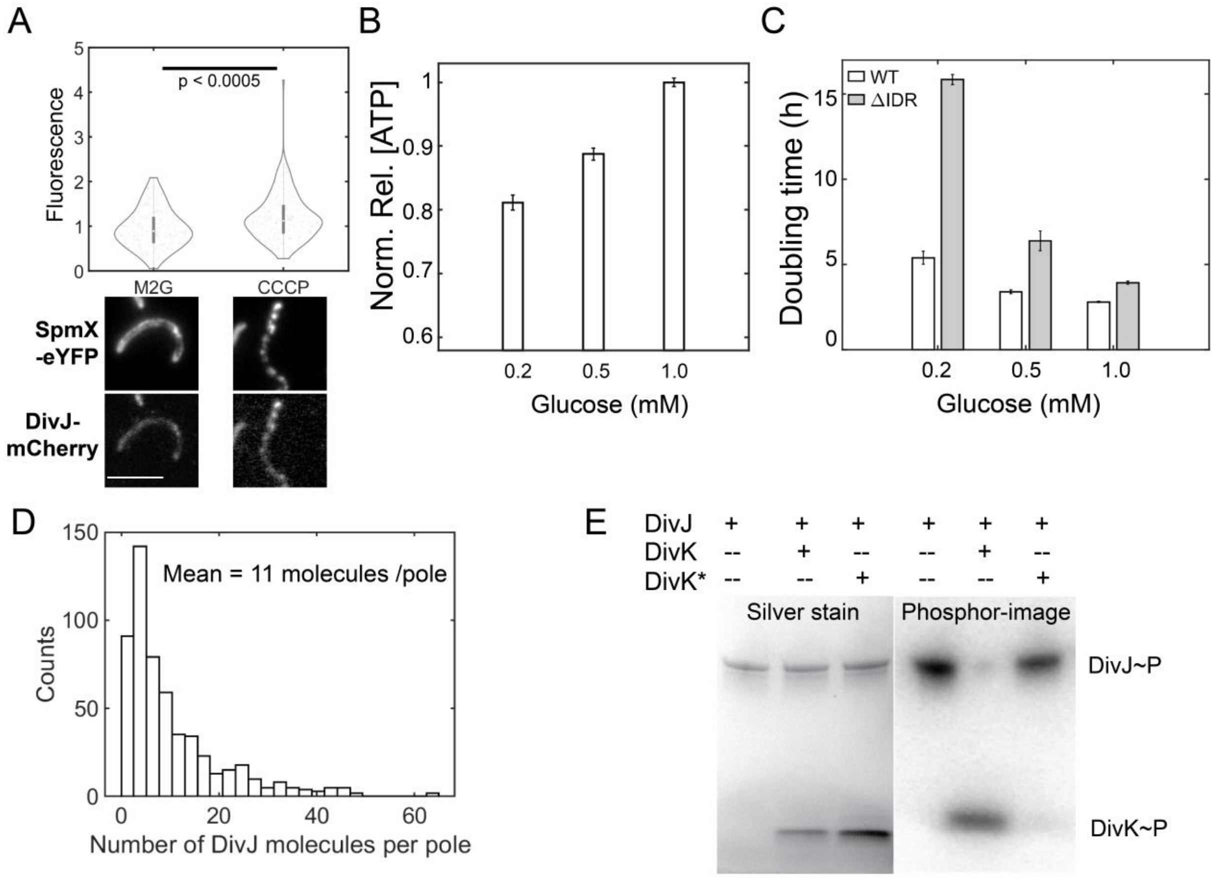

Fig. S4. Effect of ATP on DivJ localization and phenotype. (A) Distribution of the ratio of polar localized to diffuse DivJ signal in *Caulobacter* cells co-expressing SpmX-eYFP and DivJ-mCherry in a strain harboring *popZ* deletion under the control of Xylose and Vanillate promoters, respectively. DivJ-mCherry was expressed using 50 mM Vanillate while SpmX-eYFP cluster formation was induced by the addition of 0.3% Xylose for 4 hours. Cells were treated with DMSO (M2G) or 100 µM CCCP dissolved in DMSO (10 mins) followed by imaging. Representative fluorescence micrographs of cells in both eYFP and mCherry channels are shown below (scale bar is 5 µm). ~400 cells were analyzed for each condition. A two sample t-test was applied to measure statistical significance between the two distributions. Polar localization of DivJ is enhanced under ATP depletion. **(B)** Bar plot showing the relative intracellular ATP concentrations in *Caulobacter* cells grown in M2 minimal media as a function of glucose concentration from 0.2 mM to 1 mM. ATP concentrations were measured using a commercial Luciferase based luminescence assay. Error bars represent standard error of the mean from 3 biological replicates each assayed in triplicate. **(C)** Cell growth assayed through doubling time as a function of glucose in the growth

medium for WT and SpmXΔIDR cells. Glucose concentration in M2 media is denoted on the abscissa. Doubling times were calculated by averaging at least three time lags during which the optical density measured at 600 nm doubled in the exponential growth phase. Each measurement was performed in triplicate. Error bars represent standard error of the mean from three biological replicates. SpmXΔIDR cells exhibited a slower growth rate compared to WT cells as glucose was depleted. **(D)** Distribution of the number of DivJ molecules observed at the pole in a mixed population of *Caulobacter crescentus*. These distributions were obtained from wide-field fluorescence images. Samples were imaged continuously until the fluorescence signal bleached down to single molecule level. The intensity at the pole in the first diffraction limited image is divided by the mean intensity from a distribution of single eYFP molecules at the same laser power. A 31% correction based on the cell doubling time and eYFP maturation time was applied to account for immature eYFP species. **(E)** *In vitro* phosphorylation measurement of the cytoplasmic domain of DivJ (5 µM) alone, in the presence of DivK (5 µM), or DivK* (DivK(D53N)) (5 µM) incubated with 1 µCi ATP(γ–^{32}P) in a physiological buffer (50 mM HEPES-KOH, pH 7.4, 0.1 M KCl, 5 mM $MgCl_2$) for 5 mins. Phosphorylation reactions were quenched by addition of SDS, and proteins were separated via SDS-PAGE. (left) Silver-stained SDS-PAGE gel with DivJ, DivJ and DivK or DivJ and DivK* (Right) Radiograph showing the phosphorylation levels of the respective proteins from the gel on the left. The cytoplasmic domain of DivJ is capable of autophosphorylation and phosphor-transfer to DivK, but not a mutant of DivK that lacks its phospho-acceptor amino acid residue (D53).

Supplementary tables

**Table S1. Plasmids and strains used in the study**		
**A. Plasmids**		
pXYFPC-2	pxyl:eYFP *Caulobacter* integrating plasmid	(*40*)
pXYFPC-4	pxyl:eYFP *Caulobacter* integrating plasmid	(*40*)
pVCHYC-2	pvan:CHY Caulobacter integrating plasmid	(*40*)
pMCS-4	*Caulobacter* integrating plasmid	(*40*)
pMCS-6	*Caulobacter* integrating plasmid	(*40*)
pNPTS-138	*Caulobacter* counter selection plasmid	(*40*)
pAP510	pspmX:spmX-dL5	(*22*)
pAP515	pdivJ:divJ-dL5	(*22*)
pSS083	pdivJ:divJ-HaloTag	This work
pSS225	pdivJ:divJ-HaloTag-CoilY	This work
pTS35	spmX(1-162)-eYFP-CoilZ-spmX(356-431)	This work
pTS18	pdivJ:divJ-eYFP	This work
pTS23	spmX(1-162)-eYFP-spmX(356-431)	This work
pEvol-pAzF	Plasmid to incorporate p-Azido-L-phenylalanine	Addgene (*43*)
pSS47	pet28a-popZ-TEV-His6	This work
pSS121	pet28a-spmX(1-355)-F338AzF-TEV-His10	This work
AP434	pTev5-divJ(D1-187)-His6	(*8*)
pSS102	pet28a-popZ(1-102)TEV-His6	This work
pSS103	pet28a-popZ(103-177)TEV-His6	This work

pSS120	pet28a-spmX(1-155)-TEV-His10	This work
pSS165	pet28b-spmX(Δ1-155)eYFP-(156-355)-TEV-His10	This work
pAP549	pXyl:spmX	(*8*)
pAP519	pBX:spmX-eyfp	(*8*)
pTC276	pNTS138-upstream divK-divK-Halo-3xFlag-downstream divK	This work
pTC8	pBX-mCherry-popZ	This work
pAP565	pspmX:spmx(1-155)-dL5	(*22*)
pSS306	pV:divJ-mCherry	This work
pSS194	pspmx:spmX-PAmKate	(*51*)
pSS319	pBX-DivJ-eYFP	This work
B. Bacterial **strains**		
AP414	pspmX:spmX-eYFP	(*8*)
AP369	ΔspmX	(*8*)
AP510	pspmX:spmX-dL5	(*22*)
SS087	pdivJ:divJ-HaloTag	This work
TS4	pdivJ:divJ-HaloTag, pspmX:spmX-dL5	This work
TS5	ΔspmX, pdivJ:divJ-HaloTag	This work
TNC17	pBX-mCherry-PopZ	This work
TS15	pdivJ:divJ-eYFP	This work
SS297	ΔpopZ pBXyl-SpmX-eYFP	This work
TS34	ΔspmX pspmX:spmX(1-162)-eYFP-spmX(356-431)	This work

SS057	pet28a-PopZ-TEV-His6	This work
SS150	pXyl-ArcbTM2-HaloTag	This work
SS159	ΔSpmX-pXyl-ArcBTM2-HaloTag	This work
AP451	pXyl-CreS-eYFP	(*22*)
JS51	pranseE:RnaseE:eYFP	(*56*)
SS311	ΔpopZ pBXyl-SpmX-eYFP pVDivJ-mCherry	This work
SS235	ΔspmX pspmX:spmX(1-162)-eYFP-CoilZ-spmX(356-431) ΔdivJ pdivJ:divJ-HaloTag-CoilY	This work
SS324	ΔspmX pBX-DivJ-eYFP	This work
SS294	ΔspmX pspmX:spmX(1-162)-eYFP-spmX(356-431), pdivK:divK-HaloTag-3xFlag	This work
SS295	ΔspmX pspmX:spmX-dL5, pdivK:divK-HaloTag-3xFlag	This work
C. Primers		
TC29F	gagttttggggagacgaccatatgGTGAGCAAGGGCGAGGAGGATAAC	
TC30R	ggttcttgagactgatcggacatGGTACCATGCATATTAATTAAGGCGCC	
TC532F	CTTCGTCGTAATTGCCGGGATTGG	
TC533R	TCGTTGTCGTCGACGATCAGCACC	
TC540F	agctacgtaatacgactcactagtGGGTCGACAGGTCGGCCAGGGC	
TC541R	ttaaggtaccTGCAGGCTGCCTTTCCAGCAGG	
TC544F	acgatgacaagGCATGAGCGCCCGGATCCTCG	
TC545R	gtcacggccgaagctagcgaattcCTTGACGCGCGCGGACAGTTCC	
SS013F	ctttaagaaggagatatacatggctatgtccgatcagtctca	

SS014R	ggagctcgaattcggatctcagtggtggtggtggtggtgctccgtGCTCTGAAAATACAGGTTTTCgtaggcgccgcgtccccga
SS7F	tctagaaataattttgtttaactttaagaaggagatatacATGAAACCGCGTCATCAGGT
SS8R	cgacggagctcgaattcgtcagtgatggtggtggtggtgatggtgatggtgGCTCTGAAAATACAGGTTTTCCTCCACCAGCGGCACGTC
SS015F	ctttaagaaggagatatacatggctatgtccgatcagtctca
SS016R	gagctcgaattcggatctcagtggtggtggtggtggtgctccgtGCTCT
SS017F	actttaagaaggagatatacgacgaagtcgccgagcagctggtcggcgtt
SS018R	gcaagcttgtcgacggagctcgaattcggatctcagtggtggtggtggtggtgctccgtGCTCT
SS9F	tctagaaataattttgtttaactttaagaaggagatatacATGAAACCGCGTCATCAGGT
SS10R	cgacggagctcgaattcgtcagtgatggtggtggtggtgatggtgatggtgGCTCTGAAAATACAGGTTTTCCCATTCGCCGTTGGCCGG
SS321F	aaccacgatgcgaggaaacgcatatgTtgGAATTCGAAACGCTTCC
SS322R	cggagctcgagatcttaaggtaccGCGCGGCGCAAAGGCGATGACG

**Table S2. Filters used for diffraction-limited wide field imaging**		
Fluorophore	Filter (Excitation | Dichroic | Emission)	Supplier
CFP	436/20 | 455 | 480/40	Semrock
Atto 488	480/40 | 505 | 527/30	Semrock
eYFP	500/20 | 515 | 535/30	Semrock
mCherry/ Cy3/ JF549	554/23 | 573 | 609/54	Semrock
dL5/ Cy5/ JF646	635/18 | 652 | 680/42	Semrock

Movie Captions

Movie S1

Three dimensional widefield fluorescence images of condensates formed by eYFP fusion of the SpmX-IDR. 5 µM eYFP-tagged SpmX-IDR was incubated in a physiological buffer (50 mM HEPES-KOH, pH 7.4, 0.1 M KCl) for 30 mins followed by microscopy. Slices from 0-0.8 µm show the presence of collapsed eYFP-IDR condensates on glass surface treated with aminosilane. Slices above 1 µm reveal the presence of spherical condensates diffusing in the buffer.

Movie S2

Sequential two-color, three dimensional, widefield fluorescence imaging of condensates formed by eYFP-SpmX-IDR and DivJ. 5 µM eYFP-tagged SpmX-IDR was incubated in the presence of 5 µM DivJ (ΔTM, 1% labeled with JF646) in a physiological buffer (50 mM HEPES-KOH, pH 7.4, 0.1 M KCl) for 30 mins followed by microscopy. The SpmX and DivJ channels are separated in time by 8 ms. DivJ is enriched within SpmX-IDR condensates *in vitro*.

Movie S3.

3 dimensional confocal imaging of PopZ condensates at room temperature. 5 µM PopZ (1% labeled with Atto488) was reconstituted in a physiological buffer (50mM HEPES-KOH, pH 7.4, 0.1 M KCl) in an aminosilane treated glass bottom chamber for 30 mins. PopZ exhibited an arrested, beads on a string-like morphology that formed an extended network of condensates in 3 dimensions. The 3D stack was acquired using a 63x oil objective with 280 nm slices.

Movie S4.

3 dimensional images of PopZ were acquired as in Movie S3 followed by deconvolution in Fiji. A 12 µM by 12 µM by 6 µM volume was selected from a Z-stack and exported into Imaris. A Gaussian filter spanning 1 pixel was applied to the image and 3D reconstruction was performed. Various perspectives of the condensates were then exported into a movie.

Movie S5.

Observation of dynamic hollow condensates of 5 µM SpmX (ΔTM, 5% labeled with Cy3) incubated in the presence of 1 mM ATP in a physiological buffer (50 mM HEPES-KOH, pH 7.4, 0.1 M KCl, 5 mM MgCl2) at room temperature for 30 minutes. Images were captured every 10 seconds and the movie was recorded for 25 minutes. The jitter in the hollow condensates is due to bubble fusion events along the z-axis (in the plane of the image).
